# A comprehensive phylogeny of auxin homeostasis genes involved in adventitious root formation in carnation stem cuttings

**DOI:** 10.1371/journal.pone.0196663

**Published:** 2018-04-30

**Authors:** Ana Belén Sánchez-García, Sergio Ibáñez, Antonio Cano, Manuel Acosta, José Manuel Pérez-Pérez

**Affiliations:** 1 Instituto de Bioingeniería, Universidad Miguel Hernández, Elche, Spain; 2 Departamento de Biología Vegetal (Fisiología Vegetal), Universidad de Murcia, Murcia, Spain; Iwate University, JAPAN

## Abstract

Understanding the functional basis of auxin homeostasis requires knowledge about auxin biosynthesis, auxin transport and auxin catabolism genes, which is not always directly available despite the recent whole-genome sequencing of many plant species. Through sequence homology searches and phylogenetic analyses on a selection of 11 plant species with high-quality genome annotation, we identified the putative gene homologs involved in auxin biosynthesis, auxin catabolism and auxin transport pathways in carnation (*Dianthus caryophyllus* L.). To deepen our knowledge of the regulatory events underlying auxin-mediated adventitious root formation in carnation stem cuttings, we used RNA-sequencing data to confirm the expression profiles of some auxin homeostasis genes during the rooting of two carnation cultivars with different rooting behaviors. We also confirmed the presence of several auxin-related metabolites in the stem cutting tissues. Our findings offer a comprehensive overview of auxin homeostasis genes in carnation and provide a solid foundation for further experiments investigating the role of auxin homeostasis in the regulation of adventitious root formation in carnation.

## Introduction

In many plant species, the vegetative propagation of elite lines depends on the rooting ability of stem cuttings, a process affected by complex interactions between nutrients and phytohormone levels, in which auxin plays an essential role [1, 2]. Carnation (*Dianthus caryophyllus* L.) is the fifth most important ornamental plant species worldwide [3]. To deepen our understanding of the regulatory events underlying adventitious root formation in carnation, we studied two lines, ‘2101–02 MFR’ and ‘2003 R 8’; these lines were selected because of differences in their rooting performances from the rooting performance of the ‘Master’ reference cultivar [4, 5]. We characterized gene expression profiles and functional changes during the early stages of adventitious rooting in the stem cuttings of these two cultivars, providing a number of molecular, histological and physiological markers to initiate the genetic dissection of adventitious root formation in this species [6, 7].

**Fig 1:**
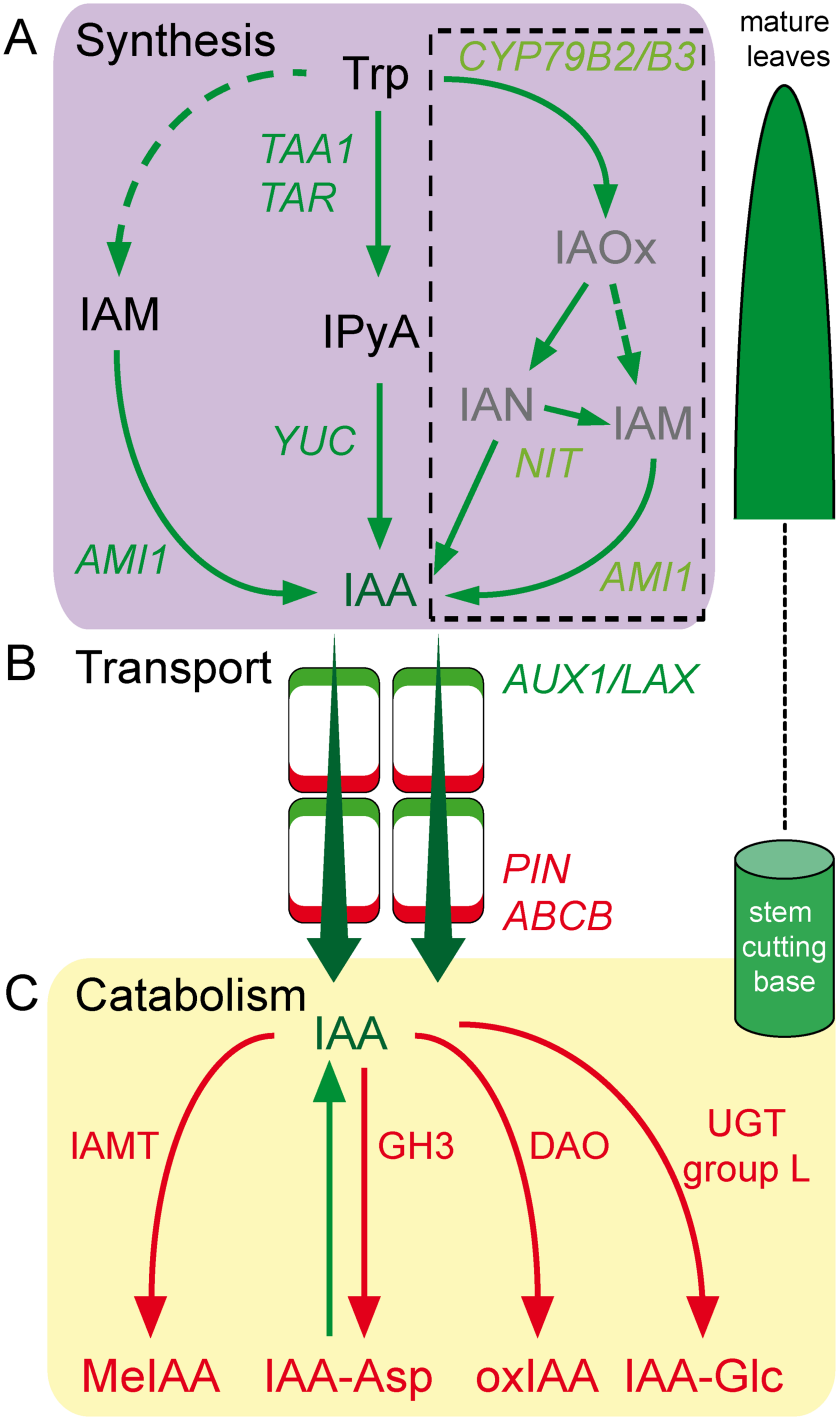
Proposed pathways of IAA homeostasis in carnation stem cuttings. (A) Auxin biosynthesis. In the middle box, the IPyA pathway is shown, while the IAM pathway is shown in the left-side box. The IAOx pathway, which is restricted to indole-glucosinolate-producing plant species such as *Arabidopsis thaliana*, *Brassica napus* or *Sinapis alba*, is given in the right-side box. (B) Auxin transport. (C) Auxin catabolism. Dashed lines indicate assumed reaction steps for which the corresponding enzymes have yet to be identified. Proteins are abbreviated as follows: ABCB, ATP-binding cassette transporter subfamily B; AMI1, amidase 1; AUX1/LAX, AUX1 and LAX auxin influx carriers; CYP79B2/B3, cytochrome P450 monooxygenase 79B2/B3; DAO, dioxygenase for auxin oxidation; GH3, GRETCHEN HAGEN3 acyl amido synthase; IAMT, indole-3-acetic acid methyltransferase; NIT, nitrilase; PIN, PIN-FORMED auxin efflux facilitators; UGT (group L), UDP glucosyltransferases; TAA1, tryptophan aminotransferase of Arabidopsis 1; TAR, tryptophan aminotransferase-related; YUC, YUCCA. Metabolites are abbreviated as follows: IAA, indole-3-acetic acid; IAA-Asp, indole-3-acetyl-aspartic acid; IAA-Glc, 1-O-(indol-3-ylacetyl)-β-D-glucose; IAM, indol-3-acetamide; IAN, indole-3-acetonitrile; IAOx. indole-3-acetaldoxime; IPyA, indole-3-pyruvic acid; MeIAA, methyl-IAA ester; oxIAA, oxindole-3-IAA; Trp, tryptophan.

Indole-3-acetic acid (IAA) is the main endogenous form of active auxin, which is primarily synthesized in leaves [8–10]. Multiple pathways have been postulated to contribute to *de novo* auxin biosynthesis in plants (Fig 1A). IAA biosynthesis can occur through two routes: tryptophan (Trp)-dependent and Trp-independent pathways [11]. The conversion of Trp to IAA occurs via different pathways named after its downstream intermediate, namely, the indole-3-acetaldoxime (IAOx), indole-3-pyruvic acid (IPyA) and indol-3-acetamide (IAM) pathways [9, 11]. Thus far, the endogenous IAOx pathway has only been identified in some Brassicaceae species [12] indicating that this pathway is not common in plants and is absent in carnation.

Auxin transport occurs either via long-distance transport through the phloem or polar cell-to-cell transport mediated by auxin carriers at the plasma membrane (Fig 1B) [13]. The latter is mediated by three gene families, with the AUXIN RESISTANT1/AUX1-LIKE (AUX/LAX) family coding for auxin influx proteins and the PIN-FORMED (PIN) family and some ATP-binding cassette transporter subfamily B (ABCB) members coding for auxin efflux/conditional transporter proteins [14]. In addition, the endogenous auxin pool includes active auxin molecules and a mixture of modified inactive auxins and precursors [9, 15]. A wide range of molecules that respectively conjugate amino acids or carbohydrates to auxin have been identified (Fig 1C), such as GRETCHEN HAGEN3 (GH3) acyl amido synthases group II [16, 17], UDP glucosyltransferases (UGT) UGT74D1 and UGT84B1 members [18, 19]. Recently, it has been reported that the Arabidopsis DIOXYGENASE FOR AUXIN OXIDATION1 (DAO1) acts in concert with GH3 to maintain the optimal level of inactive auxins, such as oxindole-3-IAA (oxIAA) or indole-3-acetyl-aspartic acid (IAA-Asp) [20, 21]. On the other hand, diverse studies have suggested that the conversion of active IAA to inactive methyl-IAA ester (MeIAA) is driven by the enzymatic action of IAA CARBOXYMETHYLTRANSFERASE1 (IAMT1) [22, 23]. In turn, these auxin storage forms might regulate auxin homeostasis (and hence its function) during plant growth and development [24].

Comparative genomic analysis is a useful approach to understand the molecular basis of auxin homeostasis, unravel potential roles for specific auxin biosynthetic pathways and obtain information about their evolutionary relationship as regards adventitious root development. Recently, similar analyses have been performed with other gene families in plants [25–27]; which resulted in adding of valuable information about the identification and annotation of new genes, deciphering gene evolutionary processes and comparing tissue-specific expression patterns in different species. By using phylogenetic analyses, we identified and properly annotated functional genes in the carnation genome whose products participate in auxin transport, auxin biosynthesis and auxin catabolism. Additionally, previous RNA-seq data obtained in our laboratory [6] was used to confirm gene structure as well as to study the expression profiles of these genes during rooting in stem cuttings from two carnation cultivars with different rooting behaviors, ‘2101–02 MFR’ and ‘2003 R 8’. These results allowed us to suggest that differential auxin homeostasis between cultivars might regulate adventitious rooting differences in carnation stem cuttings.

## Materials and methods

### Identification of auxin homeostasis gene and protein families

The identification of putative homologs of genes encoding proteins involved in auxin biosynthesis, auxin transport and auxin catabolism was performed by in *silico* analyses. The gene and protein families selected in this study were: (1) ATP-binding cassette (ABC) transporters of the B class (ABCB), AUXIN RESISTANT1/LIKE AUXIN1 (AUX/LAX) and PIN-FORMED (PIN) from auxin transport; (2) AMIDASE1 (AMI1), TRYPTOPHAN AMINOTRANSFERASE OF ARABIDOPSIS1 (TAA1), TRIPTOPHAN AMINOTRANSFERASE RELATED (TAR) and YUCCA (YUC) from auxin biosynthesis; and (3) DIOXYGENASE FOR AUXIN OXIDATION (DAO), GRETCHEN HAGEN3 (GH3), INDOLE-3-ACETIC ACID METHYLTRANSFERASE1 (IAMT1) and UDP-GLYCOSYLTRANSFERASES (UGT) (group L) from auxin catabolism.

Using the full-length protein sequences of *Arabidopsis thaliana* as queries, we carried out BLAST searches against the corresponding reference genomes from twelve plant species (S1 File), including two monocots (*Brachypodium distachyon* and *Oryza sativa*), nine dicots (*Arabidopsis thaliana*, *Cucumis melo*, *Dianthus caryophyllus*, *Eucalyptus grandis*, *Fragaria vesca*, *Medicago truncatula*, *Populus trichocarpa*, *Solanum lycopersicum* and *Vitis vinifera*) and a lycophyte (*Selaginella moellendorffii*), which was used as an outgroup [28]. BLAST searches were performed against the NCBI (https://blast.ncbi.nlm.nih.gov/Blast.cgi), Carnation DB (*Dianthus caryophyllus*, http://carnation.kazusa.or.jp/) and Sol Genomics Network (*Solanum lycopersicum*, https://solgenomics.net/) databases. Putative homologs (S1 File) were first selected based on protein query cover and e-value parameters and were used for the subsequent phylogenetic analysis.

### Phylogenetic analysis and gene structures in carnation

The multiple-sequence alignments of the full-length protein sequences selected in each protein family were retrieved using the MUSCLE tool in the MEGA 7 package [29]. Aligned protein sequences were trimmed for unaligned residues within regions displaying the highest variability. In each protein family, we searched the evolutionary model that best fit the alignments using the ‘Find Best DNA/Protein Models’ tool in the MEGA7 package. Phylogenetic trees were constructed by using the Maximum Likelihood method with a bootstrap analysis of 100 replicates. The substitution model was adjusted depending on the protein family analyzed, as follows: LG was used to analyze the AMI1 family; LG Gamma Distributed with Invariants sites was used to analyze the ABCB, AUX/LAX, TAA/TAR, UGT (group L) and YUC families; LG Gamma Distributed was used to analyze the PIN family; and JTT with Gamma Distributed used to analyze the DAO, GH3 and IAMT families. Phylogenetic trees were drawn in MEGA 7 [29]. The unique identification numbers for the protein sequences included in the phylogenetic analysis are listed in S1 File.

Gene structure models were constructed based on previous reannotations of the carnation reference genome [6, 30]. Genes were visualized with Integrative Genomics Viewer Software [31, 32], and individual sequencing reads were aligned using TopHat [33].

### RNA-sequencing (RNA-seq) and gene expression profiles in carnation stem cuttings

Sample collection and RNA extractions were performed as previously described [6]. Briefly, stem cutting samples were freshly collected at the rooting station (-23 h after planting), wrapped in plastic bags after pinching and sent to the laboratory while refrigerated in complete darkness (-15 h after planting). Next, the stem cutting bases were submerged for 15 h in a water solution with or without auxin, and then cuttings were individually planted in moistened perlite plugs (0 h after planting). The basal regions of the stem cuttings were collected at 0, 6, 24 and 54 h after planting. The frozen stem cutting bases were submerged in liquid nitrogen and squeezed with a mortar and pestle. Total RNA from the powder tissue was extracted using a Spectrum^™^ Plant Total RNA kit (Sigma-Aldrich, USA).

RNA-sequencing (RNA-seq) experiments were performed by Macrogen, Korea. To visualize the gene expression profiles, we constructed heat maps using the normalized reads for genes of interest, as previously described [6].

### Phytohormone extraction and analysis

Phytohormones were extracted and analyzed as described elsewhere [6]. Briefly, ~120 mg of frozen tissue was extracted twice with 1 ml of 80% methanol/water and centrifuged at 20,000 *g* for 15 min at 4°C. The supernatant was passed through a C18 cartridge, and the samples were collected in a 5-ml tube for speed-Vac evaporation to dryness. The residue was resuspended in 1 ml of 20% methanol/water. Ten μl of filtrated extract as injected in a U-HPLC-MS system consisting of an Accela Series U-HPLC (ThermoFisher Scientific, USA) coupled to an Exactive mass spectrometer (ThermoFisher Scientific) using heated electrospray ionization (HESI) interface. Mass spectra were obtained using Xcalibur software version 2.2 (ThermoFisher Scientific). The auxin homeostasis metabolites were identified according to the molecular mass and retention time values in the total ion chromatograms from the phytohormone analysis. In each compound, the area obtained was used for comparisons.

## Results and discussion

### Identification of genes involved in auxin biosynthesis in carnation stem cuttings

In this work, orthologous genes coding putative auxin biosynthetic, transport and catabolic proteins in the carnation genome were identified with previously published annotated genomes and transcriptome sequences [6, 30] by the following methods: i) identification of the reciprocal best-hits (RBH) between carnation and Arabidopsis protein sequences, ii) construction of maximum-likelihood trees built with homologous protein sequences of selected plant species, and iii) analysis of gene expression profiles from previous RNA-seq experiments.

#### The IPyA pathway

The IPyA pathway seems to be the main contributor to IAA synthesis in many plant species [11, 34]. In the initial step, Trp is converted to IPyA by the TRYPTOPHAN AMINOTRANSFERASE OF ARABIDOPSIS1 (TAA1) and TRYPTOPHAN AMINOTRANSFERASE RELATED (TAR) enzymes, and then the YUCCA (YUC) family of flavin monooxygenases converts IPyA to IAA [34–36]. The *TAA1*/*TAR* and *YUC* genes are widely distributed in vascular and nonvascular plants, indicating that their functions in IAA biosynthesis are evolutionary conserved in the plant kingdom [8]. Although indole-3-acetaldehyde (IAAld) has been hypothesized to be an intermediate in the IPyA pathway in plants, there is some controversy about whether IAAld contributes to this pathway *in vivo* [11].

We retrieved 62 and 135 protein sequences belonging to the TAA1/TAR and YUC families, respectively, from twelve plant species (S1 File). The putative TAA1/TAR proteins in *Selaginella moellendorffii* served as the outgroup (S1 Fig). Similar to previous results on TAA1/TAR proteins in *Arabidopsis thaliana* [37], the TAA1 and TAR1 homolog proteins grouped together, while all the TAR2 proteins grouped in a single cluster, and the TAR3 and TAR4 proteins grouped separately from the TAA1, TAR1 and TAR2 proteins (S1 Fig).

**Fig 2:**
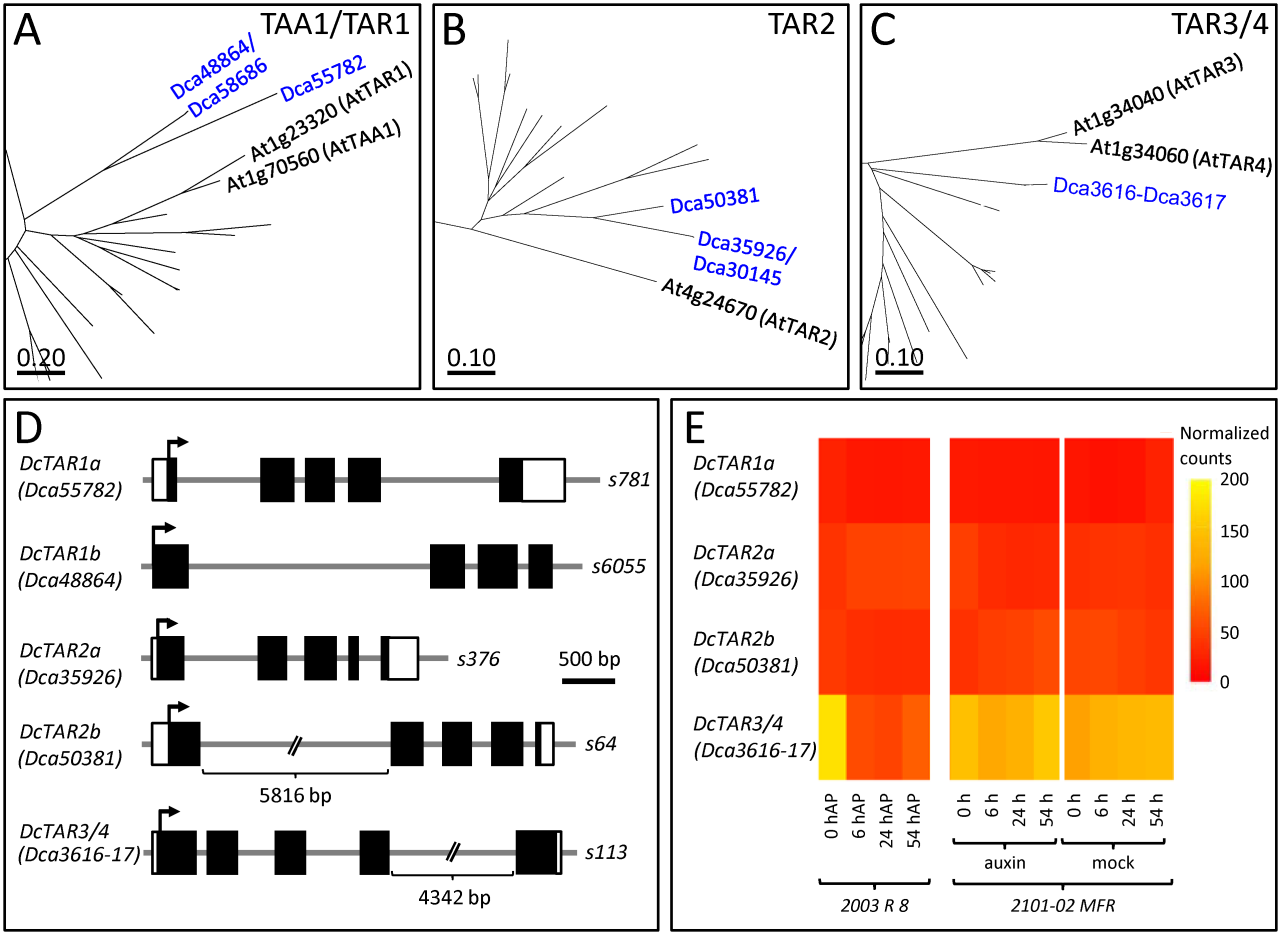
Phylogenetic analyses of the TAA1/TAR gene family in carnation. Boxes show magnifications of the tree branches containing the (A-C) TAA1/TAR protein family members studied in this work. Full phylogenetic trees are shown in S1 Fig. The evolutionary history was inferred by using the Maximum Likelihood method based on the Le Gascuel model [38]. Trees were drawn to scale, with branch lengths representing the number of substitutions per site. These analyses were conducted in MEGA7 [29], as described in the Materials and methods section. (D) Gene structure and (E) expression profiles of TAA1/TAR genes. UTR regions and exons are represented by white and black boxes, respectively; introns are depicted as gray lines. UTR, intron and exon lengths were determined using previous RNA-seq data [6].

We found three and four TAA1/TAR members in *Brachypodium* and rice respectively that grouped apart from the TAA1/TAR and TAR2 clusters in the studied dicots (S1 Fig). The TAA1/TAR complement in the dicots ranged from three members in *Cucumis sativus* to nine members in *Vitis vinifera* (S1 Fig). Although eight carnation TAA1/TAR proteins have been previously annotated [30], we suspect that some of these gene annotations are unreliable. On the one hand, by comparing the ~1.1 Kb ORFs from *Dca35926* and *Dca30145*, we found eight polymorphisms, and this number increased to 16 when 2.14 Kb of their genomic DNA were considered. These polymorphisms were randomly distributed along the DNA sequence, with no preferences for the non-expressed regions. Similar results were found for *Dca48864* and *Dca58686*. These intriguing results might be explained by the high heterozygosity of the sequenced carnation genome, which might interfere with genome annotation accuracy [30]. Hence, we excluded the *Dca30145* and *Dca58686* genes from further analyses, as we considered them to be allelic variants of *Dca35926* and *Dca48864*, respectively. On the other hand, *Dca3616* and *Dca3617* might represent two partial annotations of a single gene, which was re-named as *Dca3616-Dca3617*. Based on protein sequence similarity (Table 1A), the proteins encoded by *Dca48864* and *Dca55782* showed the highest homology with TAR1 (Fig 2A). The *Dca35926*- and *Dca50381*-encoded proteins was assigned to the TAR2 cluster (Fig 2B), while the gene encoded by *Dca3616-Dca3617* was assigned to the TAR3/TAR4 cluster (Fig 2C). The TAA1/TAR genes found in carnation had five exons (*Dca55782*, *Dca35926*, *Dca50381* and *Dca3616-Dca3617*), except for *Dca48864*, which had four. We identified the 5’UTR and 3’UTR sequences in *Dca55782*, *Dca35926*, *Dca50381* and *Dca3616-Dca3617*, as *Dca48864* was not expressed during adventitious root formation. The size of the 5’UTR regions ranged from 52 nt (*Dca35926*) to 164 nt (*Dca50381*), while the size of the 3’UTR regions varied from 127 nt (*Dca50381*) to 410 nt (*Dca55782*) (Fig 2D). The *Dca55782*, *Dca35926* and *Dca50381* genes were expressed at very low levels during adventitious rooting in the stem bases of the two studied cultivars, ‘2101–02 MFR’ and ‘2003 R 8’, and the expression levels of these genes were not affected by the cultivar or treatment (Fig 2E). On the other hand, *Dca3616-Dca3617* was expressed at low levels during rooting in both cultivars, although additional metabolic analyses will be necessary to confirm its function during rooting. Taken together, these results suggest a reduced local auxin biosynthesis in carnation stem cuttings during adventitious rooting. Based on the protein sequences, gene structures and expression levels, we renamed the carnation TAA1/TAR family genes as follows: *DcTAR1a* (*Dca55782*), *DcTAR1b* (*Dca48864* and *Dca58686*) *DcTAR2a* (*Dca35926* and *Dca30145*), *DcTAR2b* (*Dca50381*) *DcTAR3/4* (*Dca3616-Dca3617*).

**Table 1:** Auxin biosynthesis protein families in carnation.

**A. TAA1/TAR family**											
**% Identity (similarity)**	**AtTAA1**	**AtTAR1**	**AtTAR2**	**AtTAR3**	**AtTAR4**						
**Dca3616-Dca3617**	36.8 (57.8)	37.8 (58.0)	37.6 (58.4)	*50*.*0 (70*.*8)*	***50*.*8 (71*.*8)***						
**Dca35926**	*51*.*8 (69*.*6)*	49.4 (61.4)	***55*.*8 (72*.*5)***	36.5 (53.2)	36.0 (54.4)						
**Dca48864**	44.8 (63.0)	***45*.*3 (62*.*2)***	42.6 (60.6)	32.7 (52.3)	35.1 (57.6)						
**Dca50381**	43.9 (58.5)	44.1(58.3)	**52.2 (69.7)**	30.6 (46.7)	28.8 (47.4)						
**Dca55782**	33.8 (47.9)	**34.8 (48.1)**	33.6 (46.7)	27.2 (44.0)	28.1 (45.4)						
**B. YUC family**											
**% Identity (similarity)**	**AtYUC1**	**AtYUC2**	**AtYUC3**	**AtYUC4**	**AtYUC5**	**AtYUC6**	**AtYUC7**	**AtYUC8**	**AtYUC9**	**AtYUC10**	**AtYUC11**
**Dca14604**	53.1 (68.9)	52.3 (68.2)	65.8 (77.8)	56.1 (67.9)	**67.3 (82.9)**	56.6 (72.2)	64.3 (76.8)	*67*.*1 (81*.*9)*	67.1 (81.9)	40.6 (57.1)	42.1 (60.0)
**Dca24568**	49.1 (68.5)	53.9 (67.6)	*67*.*4 (81*.*7)*	52.8 (66.4)	64.1 (76.6)	53.0 (66.9)	***67*.*6 (79*.*9)***	62.7 (75.9)	62.7 (76.2)	38.9 (55.6)	40.5 (57.2)
**Dca37589**	***64*.*9 (77*.*8)***	56.1 (69.8)	53.9 (69.8)	*63*.*4 (75*.*9)*	52.9 (71.7)	54.9 (70.7)	53.4 (69.0)	52.7 (71.2)	53.9 (71.7)	39.5 (71.7)	45.4 (60.5)
**Dca38820**	51.2 (70.9)	54.7 (69.8)	66.6 (81.4)	53.5 (66.9)	65.4 (80.2)	54.4 (70.1)	**67.4 (79.4)**	63.7 (79.9)	62.5 (79.2)	39.0 (58.7)	41.6 (60.2)
**Dca41896**	54.1 (68.5)	*61*.*6 (74*.*1)*	55.5 (69.6)	53.2 (67.5)	56.0 (70.6)	***64*.*5 (76*.*9)***	54.8 (68.7)	55.5 (70.4)	56.0 (70.8)	39.5 (56.2)	41.4 (58.8)
**Dca47269**	42.2 (61.2)	39.8 (56.3)	43.8 (62.8)	43.0 (59.6)	44.0 (66.1)	38.8 (57.0)	40.4 (61.5)	42.4 (62.2)	42.7 (64.8)	***55*.*5 (71*.*4)***	*47*.*1 (67*.*2)*
**Dca53694**	49.5 (67.1)	55.2 (67.8)	42.3 (52.7)	49.2 (63.4)	42.0 (54.6)	**58.0 (70.7)**	49.5 (63.1)	42.0 (51.4)	53.3 (65.3)	36.3 (57.4)	38.5 (53.0)
**Dca56515**	52.7 (69.8)	51.5 (67.9)	66.0 (78.2)	55.0 (66.7)	***68*.*4 (83*.*6)***	55.7 (71.0)	65.6 (78.5)	66.3 (81.0)	***68*.*4 (83*.*6)***	39.8 (58.5)	41.7 (60.2)
**C. AMI1, TOC64-III and TOC64-V**											
**% Identity (similarity)**	**AtAMI1**	**AtTOC64-III**	**AtTOC64-V**								
**Dca9840**	**50.3 (57.2)**	42.1 (56.0)	48.7 (60.4)								
**Dca17854**	***63*.*0 (78*.*5)***	49.2 (58.1)	48.3 (65.3)								
**Dca19308**	32.4 (43.7)	***64*.*1 (78*.*5)***	50.9 (68.3)								
**Dca43792**	31.9 (44.3)	46.6 (62.7)	***57*.*3 (71*.*1)***								
**Dca54795**	**53.2 (67.1)**	40.5 (57.1)	40.5 (56.1)								

The highest identity value of a given carnation protein (row) is shown in bold. The highest similarity value of a given Arabidopsis protein (column) is shown in italics.

**Fig 3:**
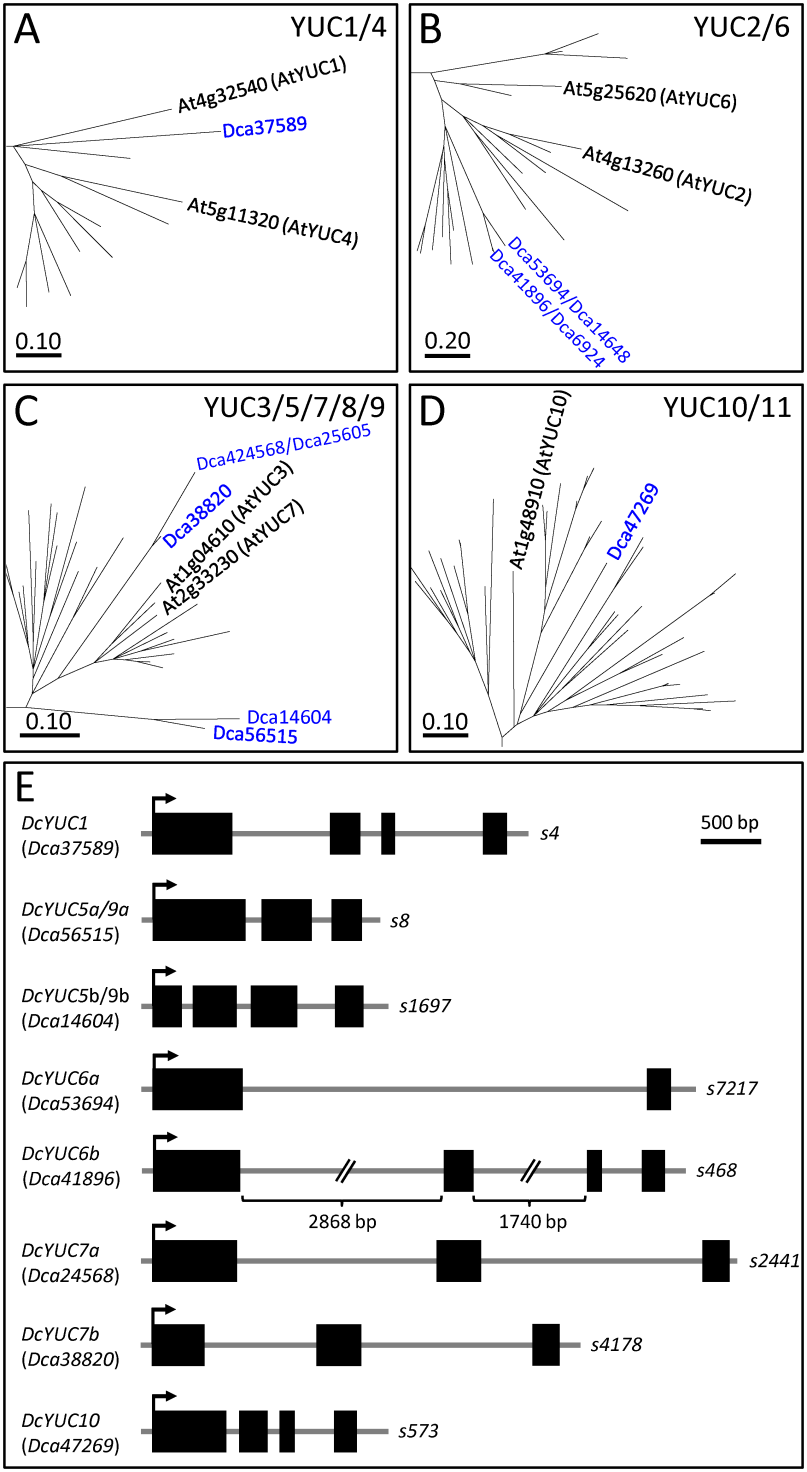
Phylogenetic analyses of the YUC gene family in carnation. Boxes show the magnification of tree branches containing the (A-D) YUC protein family members studied in this work. Full phylogenetic trees are shown in S2 Fig. (E) Gene structure of YUC genes. See legend in Fig 2 for details.

The YUC protein family in *Arabidopsis thaliana* includes 11 members that catalyze the rate-limiting step in IAA biosynthesis [34]. The phylogenetic trees grouped the YUC proteins in four clusters: YUC1/4, YUC2/6, YUC3/5/7/8/9 and YUC10/11 (S2 Fig). The YUC proteins were conserved in plants, and the number of YUC genes ranged from nine in *Cucumis melo*, *Fragaria vesca* and *Solanum lycopersicum* to 17 in *Brachypodium distachyon*. Based on protein sequence homology, we identified 11 putative *YUC* carnation genes (S1 File). The protein encoded by *Dca37589* showed the highest similarity with the AtYUC1 and AtYUC4 proteins (77.8 and 75.9% respectively) (Fig 3A and Table 1B).

Four carnation genes were assigned to the YUC2/6 cluster: the first gene pair included *Dca6924* and *Dca41896*, and the second one included *Dca14648* and *Dca53694* (Fig 3B and S2 Fig). Based on the high DNA sequence identity within 5.0 Kb windows at genomic scaffolds *s17* and *s7217*, we suspected that *Dca14648* and *Dca53694* represent two different alleles of the same gene, although we could not rule out that these two sequences arose by gene duplication. Similar results were found for *Dca6924* and *Dca41896*. The proteins encoded by *Dca24568*, *Dca25605* and *Dca38820* showed the highest similarity with AtYUC3 and AtYUC7, respectively (Fig 3C and Table 1B). Proteins putatively encoded by two other genes, *Dca56515* and *Dca14604*, were also assigned to the YUC3/5/7/8/9 cluster (Fig 3C and Table 1B). Based on DNA sequence alignment between *s2441* (*Dca24568*) and *s2450* (*Dca25605*), *Dca25605* and *Dca24568* were considered allelic variants of the same gene. Only one gene, *Dca47269*, encoded a protein that joined in the YUC10/11 cluster (Fig 3D and S2 Fig). Interestingly, none of the annotated YUC family genes described in this work (Fig 3E) were expressed during the adventitious root formation [6]. These results suggest that either auxin biosynthesis is not functional in the stem cutting base during adventitious rooting or auxin is synthesized via a non-canonical TAA1/TAR and YUC pathway in carnation stem cuttings. For future studies, we renamed the carnation YUC genes as follows: *DcYUC1* (*Dca37589*), *DcYUC2a* (*Dca53694* and *Dca14648*), *DcYUC2b* (*Dca41896* and *Dca6924*), *DcYUC3* (*Dca24568* and *Dca25605*), *DcYUC5a* (*Dca56515*), *DcYUC5b* (*Dca14604*), *DcYUC7* (*Dca38820*), and *DcYUC10* (*Dca47629*).

#### The IAM pathway

The synthesis of IAA via the IAM precursor was identified as a bacteria-specific pathway, in which the amino acid Trp is first converted to IAM by the protein encoded by the *iaaM* gene, and then IAM is converted to IAA by the protein encoded by the *iaaH* gene [39]. Although orthologues of the *iaaM* and *iaaH* genes have not currently been identified in plant genomes, the IAM precursor was detected in some plant species. The combination of homology searches, phylogenetic analyses, and biochemical assays revealed the presence of an AMIDASE1 (AMI1) enzyme in plants, which might convert IAM to functional IAA [40]. In *Arabidopsis thaliana*, the amidase family includes seven members clustered into four groups [41]. So far, the AMI1 protein in the first group is the only member of the amidase family that has been well documented as producing IAA *in vitro* [42].

**Fig 4:**
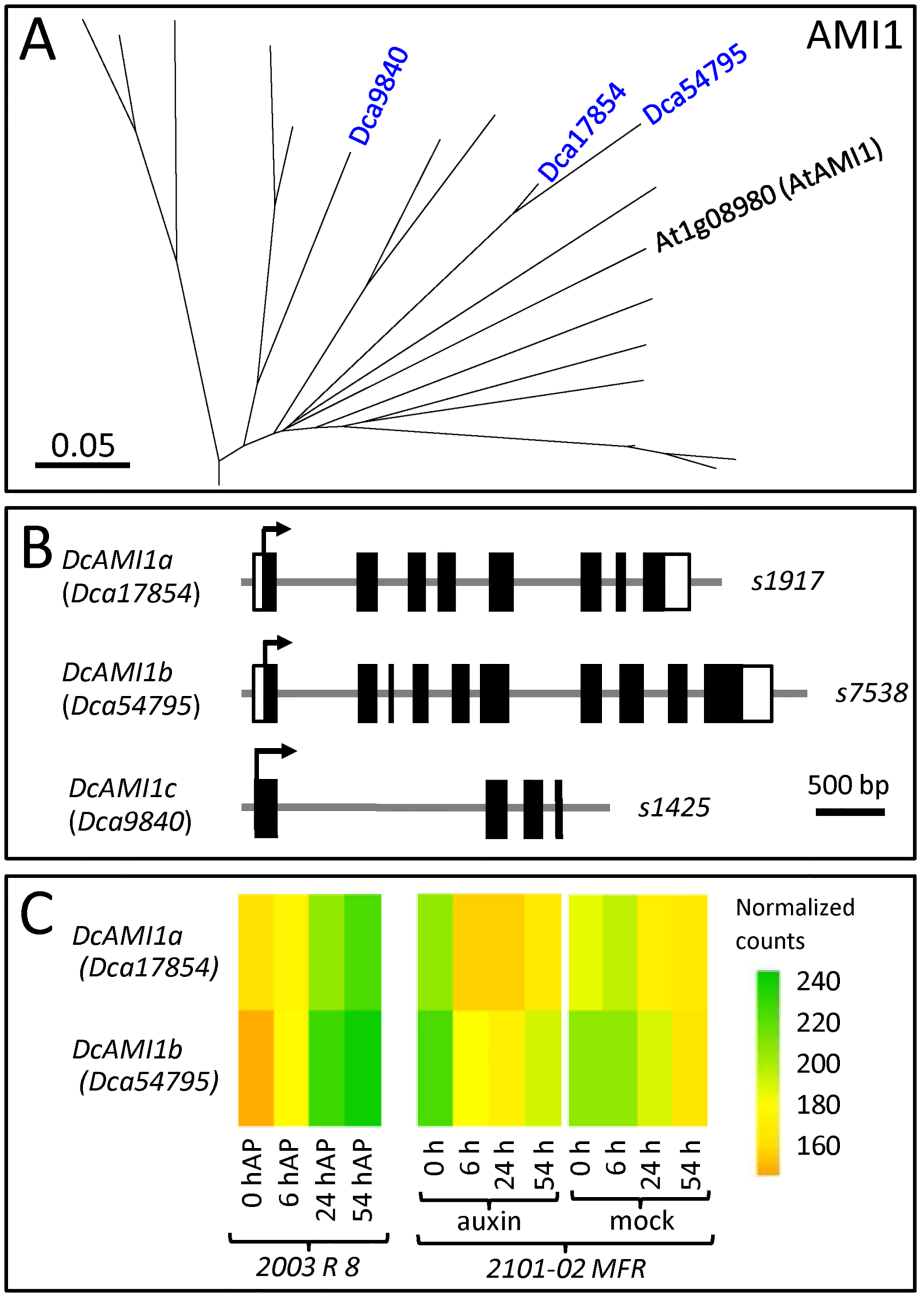
Phylogenetic analyses of the AMI1 gene family in carnation. (A) Magnification of the tree branch containing the AMI1 protein family members studied in this work. The full phylogenetic tree is shown in S3 Fig. (B) Gene structure and (C) gene expression profiles of AMI1 genes. See the legend in Fig 2 for details.

To study the amidase family, we selected 41 protein sequences belonging to the first group (S1 File), and we found they were distributed in four clusters corresponding to the *Selaginella moellendorffii* outgroup sequence and the AMI1, TOC64-III and TOC64-V sequences (S3 Fig). Three proteins grouped within the AMI1 clade, and, based on the protein sequence homology, we determined that the proteins encoded by the *Dca9840*, *Dca17854* and *Dca54795* genes share 50.3, 63.0 and 53.2% identity and 57.2, 78.5 and 67.1% similarity, respectively, with AtAMI1 (Fig 4A and Table 1C); the protein encoded by the *Dca19308* gene shared 64.1% identity and 78.5% similarity with AtTOC64-III; and the protein encoded by the *Dca43789* gene shared 57.3% identity and 71.1% similarity with AtTOC64-V (Table 1C).

Genes included in the AMI1 clade contain eight (*Dca17854*), ten (*Dca54795*) and four (*Dca9840*) exons (Fig 4B). Regarding their gene expression profiles, the lowest expression level corresponded to the *Dca9840* (*DcaAMI1c*) gene, with barely detectable normalized counts, while the *Dca17854* (*DcAMI1a*) and *Dca54795* (*DcAMI1b*) genes showed moderate expression levels during rooting in both cultivars (Fig 4C).

### Genome-wide identification of genes involved in auxin transport in carnation

#### AUX/LAX family of auxin influx carriers

*AUX/LAX* sequences have been broadly identified within Viridiplantae, which suggests a relevant role of the auxin efflux machinery in plant evolution [43]. In *Arabidopsis thaliana*, the AUX/LAX gene family includes four members (*AUX1*, *LAX1*, *LAX2* and *LAX3*) that display conserved protein sequences, gene structures and biochemical functions [44]. The AUX/LAX family is divided into two major subfamilies; one contains the *AUX1* and *LAX1* genes (AUX1/LAX1), and the other contains the *LAX2* and *LAX3* genes (LAX2/LAX3) [45].

We chose 55 protein sequences (S1 File) for the phylogenetic analysis of the AUX/LAX proteins of *Selaginella moellendorffii*; these were joined together and considered as an outgroup (S4 Fig). In *Brachypodium distachyon* and *Oryza sativa*, the AUX/LAX family proteins identified were distributed between AUX1/LAX1 and LAX2/LAX3 subfamilies with no clear distinction between the individual members included in each subfamily, mirroring the results previously found in monocots [45, 46]. Based on the protein and nucleotide sequence identities, we found evidence for recent duplication events within the AUX/LAX gene family in some species (*Cucumis melo*, *Eucalyptus grandis*, *Medicago truncatula*, *Solanum lycopersicum* and *Populus trichocarpa*). In *Solanum lycopersicum*, for example, we detected two homolog genes encoding the LAX2 protein sequence (*SlLAX2* and *SlLAX5*), while in *Populus trichocarpa*, we identified four genes in the AUX1/LAX1 group (S4 Fig). These results are in agreement with ancient tandem chromosomal duplication events within the angiosperm clade [47, 48].

**Fig 5:**
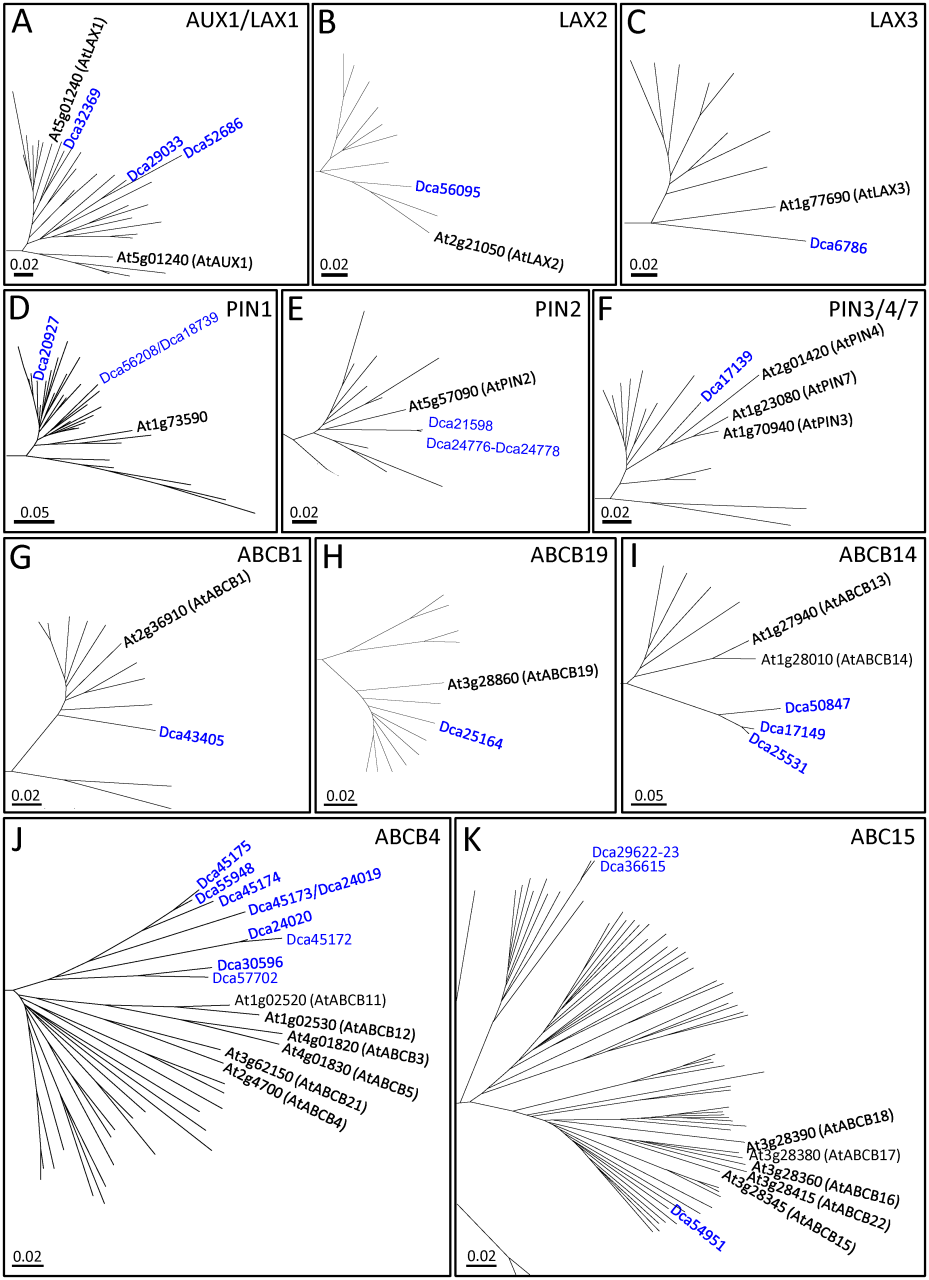
Phylogenetic analyses of (A-C) AUX/LAX, (D-F) PIN and (G-K) ABCB auxin transporter families in carnation. Full phylogenetic trees are shown in the S4 (AUX/LAX), S5 (PIN) and S6 (ABCB) Figs. See the legend in Fig 2 for details.

Based on the phylogenetic analyses, we identified five genes in the carnation genome, *Dca6786* at genomic scaffold *s127* (*s127*), *Dca29033* (*s29*), *Dca32369* (*s328*), *Dca52686* (*s698*) and *Dca56095* (*s79*), that putatively encoded AUX/LAX proteins. The proteins encoded by *Dca29033*, *Dca32369* and *Dca52686* clustered in the AUX1/LAX1 subfamily (Fig 5A). *Dca32369* is the most likely orthologue of *AtAUX1*, as the protein encoded by *Dca32369* shared 84.4% identity and 94.0% similarity with AtAUX1. *Dca29033* shared 80.3% identity and 92.6% similarity with AtLAX1, while *Dca52686* shared 77.7% identity and 92.4% similarity with AtLAX1 (Table 2A). Conversely, the proteins encoded by *Dca56095* and *Dca6786* are likely orthologues of AtLAX2 and AtLAX3, respectively (Fig 5B and 5C and Table 2A).

**Table 2:** Auxin transport protein families in carnation.

**A. AUX/LAX family**								
**% Identity (similarity)**	**AtAUX1**	**AtLAX1**	**AtLAX2**	**AtLAX3**				
**Dca6786**	78.3 (92.9)	77.3 (91.8)	80.9 (94.0)	***85*.*0 (95*.*9)***				
**Dca29033**	**80.7 (92.6)**	80.3 (92.6)	74.1 (89.7)	73.9 (89.1)				
**Dca32369**	***84*.*4 (94*.*0)***	*83*.*4 (93*.*8)*	75.9 (91.9)	72.8 (88.1)				
**Dca52686**	**78.7 (92.0)**	77.7 (92.4)	70.7 (88.9)	71.7 (87.3)				
**Dca56095**	76.0 (90.0)	74.4 (89.6)	***83*.*9 (94*.*1)***	78.3 (89.2)				
**B. PIN family**								
**% Identity (similarity)**	**AtPIN1**	**AtPIN2**	**AtPIN3**	**AtPIN4**	**AtPIN5**	**AtPIN6**	**AtPIN7**	**AtPIN8**
**Dca17139**	61.3 (72.5)	60.1 (72.6)	***76*.*9 (82*.*1)***	*73*.*9 (81*.*8)*	25.5 (33.9)	40.5 (58.1)	*75*.*2 (81*.*0)*	29.5 (39.6)
**Dca20927**	***73*.*7 (83*.*4)***	64.5 (76.8)	68.9 (79.6)	67.9 (78.6)	29.1 (23.2)	*49*.*7 (64*.*0)*	67.1 (77.0)	37.5 (51.8)
**Dca21598**	57.9 (67.6)	**72.6 (79.7)**	58.2 (68.7)	56.3 (67.8)	13.7 (34.1)	42.5 (56.1)	56.0 (67.8)	28.6 (40.6)
**Dca24776-Dca24778**	57.8 (67.6)	***71*.*8 (80*.*1)***	57.7 (68.5)	55.9 (67.0)	25.6 (34.1)	42.9 (56.2)	56.3 (68.3)	26.9 (36.4)
**Dca56208**	**66.2 (76.2)**	64.9 (75.5)	63.1 (73.6)	60.6 (70.8)	27.6 (36.8)	20.0 (24.0)	62.6 (72.7)	33.2 (45.0)
**DcPIN5 (new annotation)**	45.6 (63.7)	45.0 (65.2)	44.2 (61.8)	44.8 (63.7)	***64*.*3 (76*.*8)***	40.5 (58.1)	44.2 (62.6)	*38*.*0 (60*.*1)*
**C. ABCB family**								
**% Identity (similarity)**	**AtABCB1**	**AtABCB4**	**AtABCB14**	**AtABCB15**	**AtABCB19**			
**Dca17149**	49.0 (68.9)	41.1 (63.4)	***62*.*8 (78*.*7)***	42.1 (62.6)	50.9 (70.9)			
**Dca25164**	54.0 (73.8)	46.1 (66.9)	49.5 (69.5)	44.5 (65.9)	***88*.*4 (94*.*8)***			
**Dca25531**	48.5 (69.0)	42.2 (62.4)	**61.5 (77.8)**	42.2 (62.8)	37.8 (54.9)			
**Dca29622-Dca29623**	41.5 (61.5)	39.9 (59.9)	37.7 (58.1)	**52.1 (72.9)**	40.0 (72.9)			
**Dca30596**	46.3 (66.5)	***72*.*2 (85*.*9)***	41.5 (62.1)	42.0 (67.7)	45.2 (64.5)			
**Dca43405**	***82*.*3 (89*.*7)***	41.1 (60.3)	45.9 (63.6)	41.5 (59.7)	50.4 (68.2)			
**Dca45172**	44.3 (63.3)	**66.4 (81.9)**	41.4 (61.5)	41.7 (61.5)	45.4 (64.4)			
**Dca45173**	44.4 (65.0)	**67.5 (82.8)**	42.0 (62.2)	42.0 (63.4)	46.1 (65.7)			
**Dca45174**	45.4 (63.9)	**70.4 (84.1)**	41.7 (61.1)	42.1 (61.4)	45.8 (64.6)			
**Dca45175**	45.4 (63.1)	**61.0 (76.5)**	42.3 (62.1)	42.6 (61.7)	45.5 (64.2)			
**Dca50847**	43.3 (62.0)	36.3 (55.8)	**56.1 (70.9)**	36.6 (53.8)	44.6 (63.9)			
**Dca54951**	38.6 (55.6)	34.5 (49.9)	35.5 (49.6)	**56.5 (65.9)**	38.1 (54.3)			
**Dca57702**	46.4 (66.0)	***73*.*1 (85*.*9)***	42.1 (62.4)	42.4 (63.1)	45.2 (64.9)			

The highest identity value of a given carnation protein (row) is shown in bold. The highest similarity value of a given Arabidopsis protein (column) is shown in italics.

**Fig 6:**
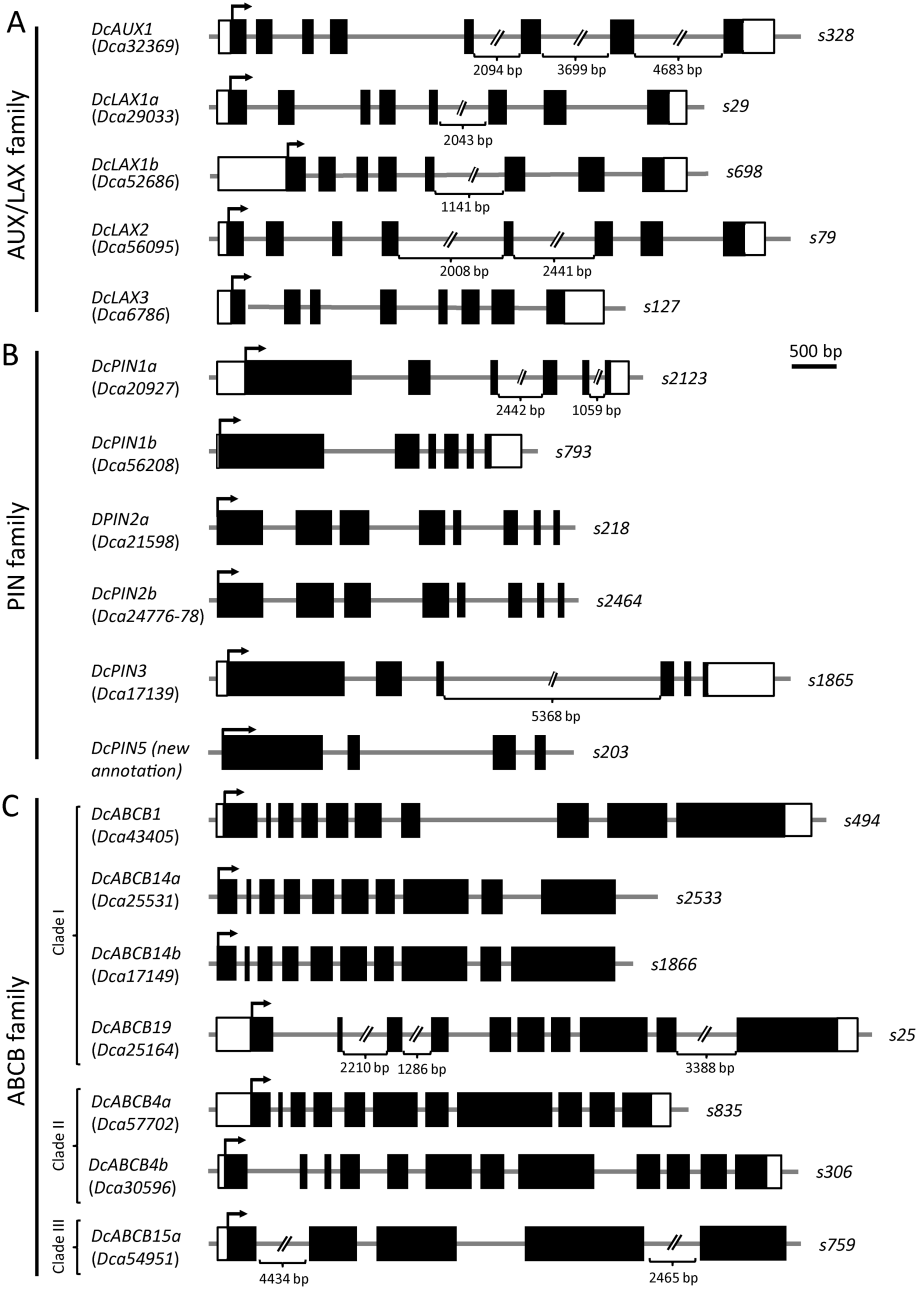
Gene structure of (A) AUX/LAX, (B) PIN and (C) ABCB auxin transporter families in carnation. See the legend in Fig 2 for details.

For the *in-silico* annotation of the AUX/LAX gene structure (Fig 6A), we used the evidence-based genome annotation described previously [6]. RNA-seq data confirmed that the carnation AUX/LAX mRNAs ranged from 1,800 nt (*Dca29033*) to 2,379 nt (*Dca52686*). *Dca52686* displayed the largest 5’ UTR region (768 nt), while the 3’ UTRs ranged from 200 nt (*Dca29033*) to 447 nt (*Dca6786*).

**Fig 7:**
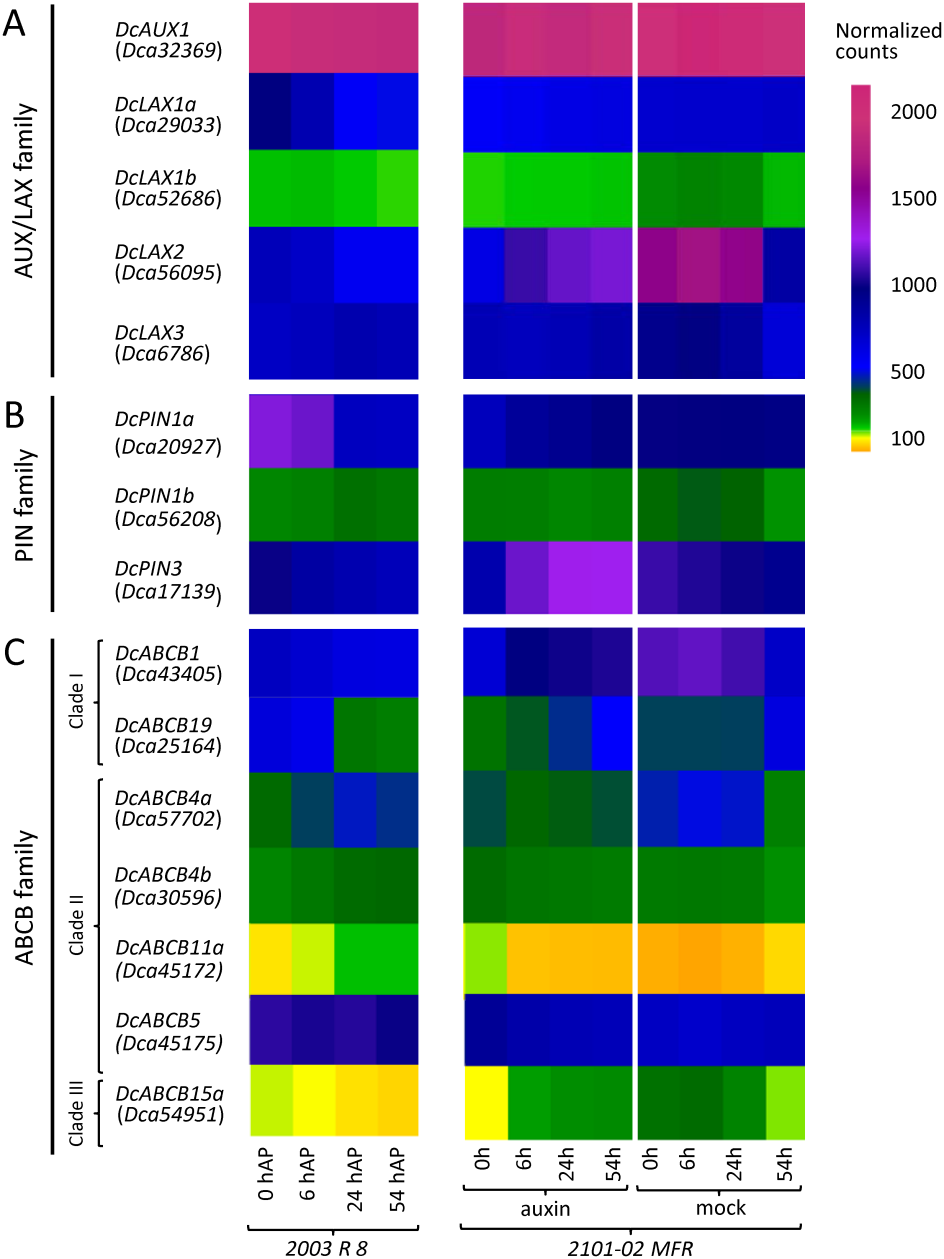
Gene expression profiles of (A) AUX/LAX, (B) PIN and (C) ABCB auxin transporter families in carnation. Only genes with detectable expression levels in previous experiments [6] were selected.

Four AUX/LAX carnation genes were expressed in the basal region of the stem cuttings of the two carnation genotypes with different adventitious root formation performances (Fig 7A). *Dca32369* was expressed at high levels in both cultivars, while the *Dca56095* expression was higher in the good-rooting cultivar (‘2101–02 MFR’) than in the bad-rooting cultivar (‘2003 R 8’). *Dca29033* and *Dca6786* were similarly expressed in both cultivars at moderate levels. *Dca52686* was expressed at low levels in both cultivars.

Based on the protein sequences, phylogeny results, gene structures and gene expression, we renamed the carnation AUX/LAX genes as follows: *DcAUX1* (*Dca32369*), *DcLAX1a* (*Dca29033*), *DcLAX1b* (*Dca52686*), *DcLAX2* (*Dca56095*) and *DcLAX3* (*Dca6786*). The high expression level of several AUX/LAX genes in the stem cutting base was consistent with the findings that polar auxin transport was required to trigger the initiation of adventitious roots in this species [4]. The dynamic expression of *DcAUX1*, *DcLAX2* and *DcLAX3* was observed in the bad-rooting cultivar ‘2003 R 8’ at the early time-points, which will require further investigation.

#### PIN family of auxin efflux facilitators

PIN-FORMED (PIN) proteins are a plant-specific family of transmembrane proteins that transport auxin in a polar manner [49–51]. PIN proteins can be divided into two broad subfamilies: (1) the canonical PINs (PIN1 to PIN4 and PIN7 in *Arabidopsis thaliana*), which are plasma-membrane localized and contain two transmembrane regions separated by a long hydrophilic loop and (2) the short-looped, endoplasmic reticulum-localized PINs (PIN5, PIN6 and PIN8).

We selected 116 PIN protein sequences (S1 File). The putative PIN proteins in the lycopod species *Selaginella moellendorffii* clustered in a separate group, as previously described [50]. The PIN proteins clustered into six different groups (S5 Fig), which were named following the different Arabidopsis PINs [51]. Within angiosperm genomes, local gene duplications and gene losses are characteristic of this gene family due to asymmetric radiations [49]. Among the studied species in this work, PIN1 and PIN5 were commonly encoded by redundant genes. However, some species seemed to have lost one or several PIN genes. This was the case for genes encoding PIN3, PIN4 and PIN6 in *Brachypodium distachyon* and *Oryza sativa*, which is consistent with previous results in other monocots [52–54]. Except for *Arabidopsis thaliana*, all studied dicot species contained one gene (*Fragaria vesca*, *Vitis vinifera*) or two genes (*Populus trichocarpa*, *Medicago truncatula*, *Solanum lycopersicum*, *Cucumis melo*, *Eucalyptus grandis*) encoding the PIN3/4/7 proteins (S5 Fig). Although the PIN protein complement was proposed to be relatively consistent among angiosperms [55], we found a striking diversity in the PIN protein number of the studied species, with *Fragaria vesca* and *Dianthus caryophyllus* genomes containing only six PIN-encoding genes each. However, we could not rule out that the small number of *PIN* genes in these two species might be due to their incomplete genome annotations.

The PIN-encoding genes identified in this work were notably different from the previously annotated PIN genes in carnation [30]. We determined that the incorrect annotation of some PIN-encoding genes caused this discrepancy. First, the *Dca24776*, *Dca24777* and *Dca24778* genes were tandemly arranged in the same region at genomic scaffold *s2464*, and their ORFs correspond to truncated versions of the same PIN protein that had been incorrectly annotated. Second, the DNA sequence of the *Dca18739* gene (*s1974*) shared 99.6% nucleotide identity with that of the *Dca56208* gene (*s793*), which is consistent with the sequence overlap between these two genomic scaffolds. Hence, we grouped and renamed *Dca24776*, *Dca24777* and *Dca24778* as *Dca24776-Dca24778*, and we excluded *Dca18739* from further analyses, as this gene is a duplicate annotation of *Dca56208* [30].

Based on phylogenetic analysis (S5 Fig), PIN1 in carnation was encoded by *Dca20927* and *Dca56208* (Fig 5D). The proteins encoded by *Dca21598* and *Dca24776-Dca24778* were included within the PIN2 clade (Fig 5E) and shared approximately 71.8% identity and 80.1% similarity with AtPIN2. The *Dca17139*-encoding protein was the only member within the PIN3/4/7 group in carnation (Fig 5F). The protein encoded by a new PIN annotation at genomic scaffold *s203* [6] shared 64.3% identity and 73.8% similarity with AtPIN5 (Table 2B). From the published draft genome sequence [30], we were not able to identify homologs of the *PIN6* and *PIN8* genes. It has been proposed that these non-canonical PIN proteins arose more recently than other PIN proteins and might have acquired novel functions in the homeostatic partitioning of auxin metabolites within the cell [49]. The intron-exon organization of *PIN* genes was highly conserved in all the studied plant species [50]. The carnation *PIN* genes were composed of six exons, except for those encoding PIN2 and PIN5 (Fig 6B). The first exon of *Dca17139*, *Dca20927* and *Dca56208* encoded approximately 65% of the corresponding PIN proteins. The same region of the PIN protein was encoded by exon 1 to exon 3 in the *Dca21598* and *Dca24776-Dca24778* genes, which both have eight exons. The size of the last four exons of all *PIN* genes was also conserved (Fig 6B), a feature that has been described for other plant genomes [50] and might represent a functional feature of *PIN* genes.

Gene expression data revealed that *Dca21598* and *Dca24776-Dca24778* were not expressed in the basal region of the carnation stem cuttings during adventitious rooting, while *Dca17139* and *Dca20927* were highly expressed in this tissue. The *Dca56208* gene displayed moderate to low expression levels in the studied dataset (Fig 7B). Notably, the expression patterns of the *Dca20927* and *Dca56208* genes were opposite in the two studied cultivars during adventitious rooting (Fig 7B). In the mock treatment, the *Dca17139* expression was similar in both cultivars, with the highest levels found at planting time (0 h after planting). Strikingly, the exogenous auxin treatment in the good-rooting cultivar ‘2101–02 MFR’ induced high expression levels of *Dca17139* (Fig 7B). We could not obtain expression data for the newly annotated *DcPIN* gene at genomic scaffold *s203* from our RNA-seq experiments, as this gene was not present in our transcriptome reannotation [6].

Based on the protein sequences, phylogeny results, gene structures and gene expression profiles, we renamed the carnation PIN-encoding genes *DcPIN1a* (*Dca20927*), *DcPIN1b* (*Dca56208* and *Dca18739*), *DcPIN2a* (*Dca21598*), *DcPIN2b* (*Dca24776-Dca24778*), *DcPIN3* (*Dca17139*) and *DcPIN5* (new annotation at *s203*). In the stem cutting base of the studied cultivars during adventitious rooting, *DcPIN1a* and *DcPIN3* were expressed at high levels, while some differences in the expression levels were found in relation to genotype and hormonal treatment.

#### ABCB family of auxin efflux carriers

Plant ATP-binding cassette (ABC) proteins, which belong to one of the largest protein families of transporters, have been classified in different subfamilies according to their structure and phylogeny [56, 57]. Among them, the ABCB subfamily of *Arabidopsis thaliana* includes 21 proteins, which are distributed in three clades and involved in a diverse range of biological processes [58]. Here, we focused our analyses on the ABCB members with functions previously linked to auxin transport, such as ABCB1, 4, 14, 15 and 19 [59, 60].

For the phylogenetic analyses of these ABCB subfamily members, we selected 187 protein sequences (S1 File). The consensus trees included all three previously described clades. The ABCB proteins of *Selaginella moellendorffii* were the outgroup (S6 Fig). In accordance with the large number of ABCB genes in other plant species [45, 57], we found 17 orthologues in *Dianthus caryophyllus*, five within clade I (ABCB1/14/19), nine within clade II (ABCB4), and three within clade III (ABCB15) (Fig 5G–5K and S6 Fig). The carnation ABCB genes contained five (*Dca54951*) to 12 (*Dca30596* and *Dca45175*) exons (Fig 6C and S7 Fig). The ABCB1 and ABCB19 clusters included two carnation genes each (Fig 5G and 5H). The protein encoded by *Dca43405* shared 82.3% identity and 89.7% similarity with AtABCB1, whereas the protein encoded by *Dca25164* shared 88.4% identity and 94.9% similarity with AtABCB19 (Table 2C). Both genes were expressed at moderate (*Dca25164*) to high (*Dca43405*) levels during rooting in the stem cuttings of both cultivars (Fig 7C). Three other carnation orthologues, *Dca17149*, *Dca25531* and *Dca50847*, showed the highest homology with the ABCB14-encoding genes in clade I (Fig 5I), but their expression was barely detectable (Fig 7C) in our RNA-seq dataset [6]. Several genes within clade II (Fig 5J) warrant further consideration. *Dca30596* and *Dca57702* shared 72.2% and 73.1% identity, respectively, with AtABCB4 (Table 2C) and were expressed at moderately high levels in the stem cutting base during adventitious rooting (Fig 7C). Based on gene structure and sequence identity (98.0%), *Dca45175* and *Dca55948* might represent two different splicing forms of the same gene, which we dubbed *DcABCB5*; this gene was highly expressed during rooting (Fig 7C). In addition, we considered *Dca24019* to be a duplicated annotation of *Dca45173*, as their sequences were 99.8% identical; we considered *Dca24020* to be a truncated reannotation of *Dca45172*, as both sequences were located in the same genomic scaffold, and their ORFs overlapped. *Dca24019* and *Dca24020* were excluded from further studies. Hence, *Dca45172* and *Dca45173* were the most homologous to ABCB11 (Table 2C), and both were expressed during rooting, albeit at very low levels. *Dca29622-23*, *Dca36615* and *Dca54951* grouped within clade III (Fig 5K), but only *Dca54951* was expressed during adventitious rooting (Fig 7C) and showed the highest homology (56.5% similarity and 65.9% identity) with AtABCB15 (Fig 5K and Table 2C). We also excluded *Dca36615* from further analyses, as we consider it a duplicate annotation of *Dca29622-23* based on 98.9% their identity at the ORF level. These results suggest that, in most cases, the non-expressed duplicated genes might function as pseudogenes, although additional studies will be required to determine whether they can be expressed in other organs and/or in response to specific conditions.

Based on protein sequence, gene structure and gene expression levels, we renamed the carnation ABCB family genes putatively linked to auxin transport *DcABCB1* (*Dca43405*), *DcABCB4a* (*Dca57702*), *DcABCB4b* (*Dca30596*), *DcABCB4c* (*Dca45174*), *DcABCB5* (*Dca45175* and *Dca55948*), *DcABCB11a* (*Dca45172* and *Dca24020*), *DcABCB11b* (*Dca45173* and *Dca24019*), *DcABCB14a* (*Dca25531*), *DcABCB14b* (*Dca17149*), *DcABCB14c* (*Dca50847*), *DcABCB15a* (*Dca54951*), *DcABCB15b* (*Dca29622-23* and *Dca36615*) and *DcABCB19* (*Dca25164*). Interestingly, two of these genes showed contrasting expression levels in the studied carnation cultivars that have different rooting performances. On the one hand, *DcABCB1* was highly expressed in the good-rooting cultivar ‘2101–02 MFR’; on the other hand, *DcABCB19* showed higher expression levels at earlier time points in the bad-rooting cultivar ‘2003 R 8’ (Fig 7C).

The *abcb1* mutants of Arabidopsis exhibit significant reductions in auxin transport and altered reproductive structures. Moreover, mutations in the ABCB19 auxin transporter in an *abcb1* mutant background showed a synergistic effect on auxin transport, enhancing the auxin deficient phenotype [61]. Functional analyses investigating the correlation between ABCB gene expression levels and polar auxin transport rates in different carnation genotypes, which is currently being performed in our laboratory, will help to understand the relevance of polar auxin transport regulation during the adventitious rooting of stem cuttings. Regarding the auxin treatment, we detected expression differences in a few genes, such as *Dca43405* (*DcABCB1*) and *Dca54951* (*DcABCB15a*). In both cases, the exogenous addition of auxin decreased the expression level of these two genes at the initial time points during adventitious rooting (Fig 7C).

### Phylogenetic analysis of genes involved in auxin catabolism in carnation

#### GH3 (group II) subfamily of auxin conjugases

*GH3* genes have been identified in many plant species, as well as in mosses and algae [62]. In *Arabidopsis thaliana*, the GH3 family includes 19 genes that are phylogenetically clustered into three groups (group I to group III) [16] and whose molecular functions have been recently clarified. Briefly, one of the two members of group I has been linked to the regulation of active jasmonate levels [63]; GH3 proteins of group II play critical roles in plant development through IAA conjugation to amino acids, such as aspartic acid (Asp) [16, 17]; and the only characterized member of the GH3 group III has been related to salicylic acid regulation and pathogen susceptibility [64].

**Fig 8:**
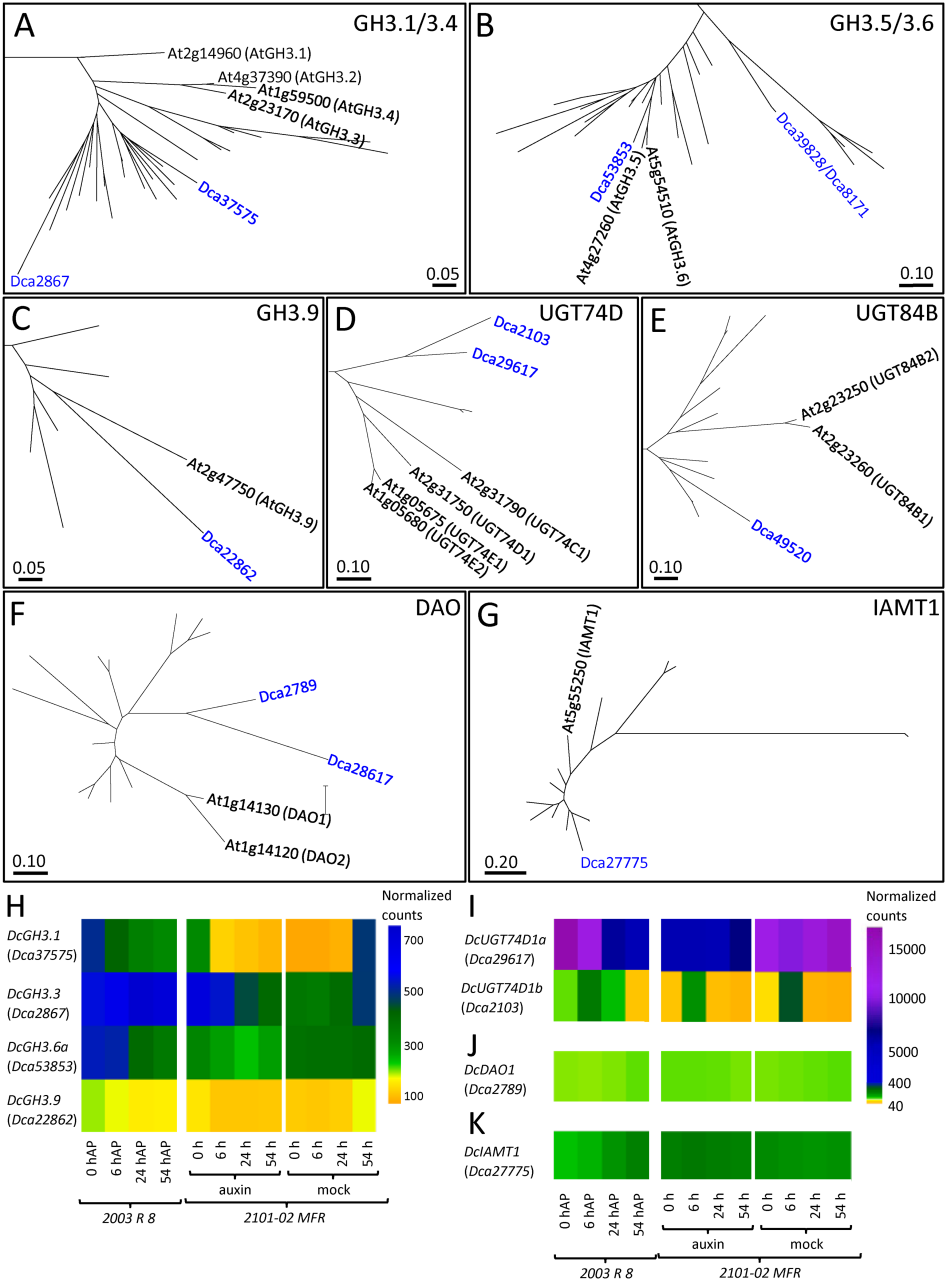
Phylogenetic analyses of (A-C) GH3 (group II), (D) UGT74, (E) UGT84, (F) DAO and (G) IAMT1 families in carnation. Full phylogenetic trees are shown in the S9 (GH3 group II), S11 (group L UGT) and S12 (DAO and IAMT1) Figs. See the legend in Fig 2 for details. (H-I) Gene expression profiles of some (H) GH3 group II, (I) UGT74, UGT84, (J) DAO and (K) IAMT1 genes are shown.

We studied 74 protein sequences of GH3 group II putatively involved in IAA conjugation (S1 File). GH3.1 to GH3.4 grouped together in one cluster, GH3.5 and GH3.6 grouped in another cluster, and GH3.9 and GH3.17 formed the third and fourth clusters, respectively (Fig 8A–8C and S8 Fig). We identified six carnation orthologues of the GH3 group II genes, namely, *Dca2867* (*s11*), *Dca8171* (*s134*), *Dca22862* (*s229*), *Dca37575* (*s4*), *Dca39828* (*s4327*) and *Dca53853* (*s727*). Sequence alignments (99.3% identity at the scaffold level and 99.5% identity at the ORF level) revealed that the *Dca8171* and *Dca39828* genes were likely the same gene and only the latter was further studied. The *Dca2867* and *Dca37575* genes were phylogenetically classified into the GH3.1/3.4 cluster (Fig 8A); *Dca39828* and *Dca53853* belonged to the GH3.5/3.6 cluster (Fig 8B); and *Dca22862* clustered to GH3.9 (Fig 8C). None of the carnation GH3 genes identified in this work clustered into the GH3.17 subgroup (S8 Fig). The proteins encoded by the *Dca37575*, *Dca2867* and *Dca22862* genes shared 80.1, 72.0 and 80.3% identity and 89.2, 73.6 and 80.3% similarity to the AtGH3.1, AtGH3.3 and AtGH3.9 proteins, respectively (Table 3A). The proteins encoded by *Dca39828* and *Dca53853* showed almost the same identity and similarity values to AtGH3.5 and AtGH3.6 (Table 3A).

**Table 3:** Auxin catabolism protein families in carnation.

**A. GH3 (group II) subfamily**								
**% Identity (similarity)**	**AtGH3.1**	**AtGH3.2**	**AtGH3.3**	**AtGH3.4**	**AtGH3.5**	**AtGH3.6**	**AtGH3.9**	**AtGH3.17**
**Dca2867**	**73.5 (83.2)**	68.3 (73.6)	72.0 (73.6)	67.6 (81.2)	63.2 (78.3)	64.4 (78.7)	52.3 (68.9)	*55*.*2 (76*.*0)*
**Dca22862**	49.6 (68.3)	*47*.*7 (76*.*0)*	*49*.*2 (76*.*0)*	48.0 (68.5)	51.1 (69.0)	50.4 (68.7)	***65*.*5 (80*.*3)***	51.6 (72.0)
**Dca37575**	***80*.*1 (89*.*2)***	*74*.*6 (76*.*0)*	*76*.*2 (76*.*0)*	*73*.*1 (83*.*9)*	67.1 (81.7)	67.3 (81.4)	51.9 (69.9)	54.7 (75.0)
**Dca39828**	63.3 (73.0)	58.6 (65.7)	60.7 (65.7)	57.1 (72.1)	73.6 (81.6)	**73.8 (81.3)**	53.3 (69.4)	57.0 (72.5)
**Dca53853**	67.2 (76.7)	64.1 (68.8)	66.0 (68.8)	61.8 (74.6)	*83*.*7 (91*.*4)*	***84*.*7 (93*.*0)***	53.2 (70.5)	55.1 (74.4)
**B. UGT (group L) subfamily**								
**% Identity (similarity)**	**AtUGT74D1**	**AtUGT84B1**						
**Dca2103**	**44.5 (62.7)**	30.4 (49.5)						
**Dca29617**	***50*.*1 (67*.*9)***	34.3 (55.7)						
**Dca49520**	34.3 (51.5)	***39*.*6 (57*.*4)***						
**C. DAO family**								
**% Identity (similarity)**	**AtDAO1**	**AtDAO2**						
**Dca2789**	***54*.*2 (68*.*8)***	*50*.*0 (66*.*3)*						
**Dca28617**	**44.2 (58.5)**	42.2 (60.1)						
**D. IAMT1**								
**% Identity (similarity)**	**AtIAMT1**							
**Dca27775**	***73*.*4 (84*.*0)***							

The highest identity value of a given carnation protein (row) is shown in bold. The highest similarity value of a given Arabidopsis protein (column) is shown in italics.

These five carnation genes from GH3 group II contained three (*Dca2867* and *Dca22862*) or four (*Dca37575*, *Dca39828* and *Dca53853*) exons (S10 Fig), and their coding region sizes ranged from 1,736 (*Dca22862*) to 1,866 bp (*Dca39828*). *Dca39828* was not expressed in our experimental dataset [6]. *Dca22862* displayed mild expression levels, and no differences were observed between cultivars, treatments or over the studied time course (Fig 8H). *Dca2867* was expressed at higher levels in the bad-rooting cultivar ‘2003 R 8’, and its expression increased significantly during rooting (Fig 8H). In the good-rooting cultivar (‘2101–02 MFR’), *Dca2867* showed the highest expression levels early after the auxin treatment (0–6 h after planting), and its expression subsequently decreased (24–54 h after planting) (Fig 8H). In the bad-rooting cultivar ‘2003 R 8’, the *Dca37575* and *Dca53853* expression levels were higher at the initial time points and progressively decreased, while an opposite expression response was observed in the mock-treated ‘2101–02 MFR’ stem cuttings (Fig 8H). It is worth mentioning that the overall expression level of these three *DcGH3* genes was considerably lower in the mock-treated ‘2101–02 MFR’ stem cuttings than in the ‘2003 R 8’ stem cuttings. Because auxin conjugation depends on GH3 protein activity, among others, our results suggest that the level of amino acid-conjugated (inactive) auxins might be lower in the ‘2101–02 MFR’ cultivar than in the ‘2003 R 8’ cultivar. Further investigations will help determine whether GH3 expression differences partially account for the rooting differences observed between these two cultivars. We renamed the GH3 group II family genes putatively involved in IAA-amino acid conjugation *DcGH3*.*1* (*Dca37575*), *DcGH3*.*3* (*Dca2867*), *DcGH3*.*6a* (*Dca53853*), *DcGH3*.*6b* (*Dca39828* and *Dca8171*), and *DcGH3*.*9* (*Dca22862*).

#### UGT (group L) subfamily of auxin glycosylases

In addition to amino acids, IAA can be conjugated to glucose (Glc) or glucuronic acid by different members of the UDP-glycosyltransferase (UGT) family. More than 100 UGT proteins belonging to 14 groups have been identified in Arabidopsis [65, 66]. Among them, group L of UGTs catalyzes the glycosylation of IAA, indole-3-butyric acid, and various aromatic compounds, such as salicylic acid, anthranilate, sinapic acid and indole-glucosinolate [19]. Only UGT74D1 and UGT84B1 activities have been previously associated with endogenous IAA catabolism [18, 19, 67]. While UGT84B1 catalyzes the conversion of IAA to 1-O-(indol-3-ylacetyl)-β-D-glucose (IAA-Glc) *in planta* [18], UGT74D1 glycosylates both IAA and its oxidized form [19, 67].

To determine the carnation genes encoding UGT74D1 and UGT84B1, we selected 75 UGT group L protein sequences from different plant species (S1 File). Our phylogeny analysis clearly identified UGT74, UGT75 and UGT84 clusters (Fig 8D and 8E and S10 Fig). Eleven, three and two different carnation proteins joined the UGT74, UGT84 and UGT75 clusters, respectively (S10 Fig). Among them, the protein encoded by the *Dca29617* gene shared 55.6% identity and 73.9% similarity to AtUGT74D1, followed by the protein encoded by *Dca2103*, which shared 44.5% identity and 62.7% similarity to AtUGT74D1 (Fig 8D and Table 3B). *Dca29617* contained two exons encoding a protein of 349 amino acids; its ORF was 1,050 bp long (S11A Fig), and its expression level and profile depended on cultivar, experimental time and treatment; the *Dca2103* expression profile depended on experimental time and cultivar but not on treatment (Fig 8I). In the ‘2003 R 8’ cultivar, higher expression levels were observed at the initial time points (0–6 h after planting), and the expression progressively decreased from 24–54 hours after planting (Fig 8I). On the other hand, the mock-treated stems of the ‘2101–02 MFR’ cultivar showed high expression levels and subtle expression changes during rooting (Fig 8I). Regarding the carnation UGT84B1 orthologues, the *Dca49520* gene was the only member identified (Fig 8E). *Dca49520* is a gene of 1,506 bp (S11A Fig) that has no introns and encodes a 501 amino acid protein sharing 43.5% identity and 57.7% similarity with AtUGT84B1 (Table 3B). The *Dca49520* gene was expressed at low levels in both of the carnation cultivars studied in this work, and no significant changes were observed in the auxin treatment (Fig 8I). Hence, we renamed *DcUGT74D1a* (*Dca29617*), *DcUGT74D1b* (*Dca2103*) and *DcUGT84B1* (*Dca49520*).

#### DAO family of auxin dioxygenases

The conversion of IAA to oxindole-3-IAA (oxIAA) plays an important role in auxin catabolism [68, 69]. AtDAO1 and AtDAO2 dioxygenases are known to be involved in the conversion of IAA to oxIAA in Arabidopsis [21, 70] and rice [71]. The disruption of AtDAO1 function causes a significant reduction in oxIAA and oxIAA-Glc, resulting in the up-regulation of *GH3* genes and thereby increasing the endogenous levels of other inactive IAA conjugates [20, 21].

Sixteen DAO homolog proteins were retrieved from the NCBI database (S1 File). We were unable to identify *AtDAO1* homologs in *Selaginella moellendorffii* and *Cucumis melo*; only one *AtDAO1* homolog was found in *Brachypodium distachyon*, *Fragaria vesca*, *Medicago truncatula* and *Vitis vinifera*; the remaining species contained two putative *AtDAO1* homologs each (S1 File and S12A Fig).

In carnation, the *Dca2789* and *Dca28617* genes were identified as putative *AtDAO* homologs (Fig 8F and Table 3C). The coding region of *Dca2789* was 891 bp long, with three exons encoding a 296 amino acid protein (S11B Fig). The coding region of *Dca28617* was 756 bp long, contained four exons and encoded a 251 amino acid protein (S11B Fig). *Dca28617* was not expressed during adventitious rooting [6], while the *Dca2789* gene was expressed in both of the studied cultivars at similar moderate levels (Fig 8J). Our expression patterns were in agreement with results in Arabidopsis, where *AtDAO2* was expressed several-fold lower than *AtDAO1* [70]. Accordingly, we renamed *Dca2789* as *DcDAO1*, and we renamed *Dca28617* as *DcDAO2*. Based on *DcDAO* expression and contrary to the case of Arabidopsis [20, 21], the conversion of IAA to oxIAA in carnation might not limit the regulation of endogenous auxin activity.

#### IAMT family

The conversion of IAA to MeIAA by IAA carboxyl methyltransferase IAMT plays an important role in modulating auxin homeostasis and controlling plant development [23]. IAMT activities have been identified in Arabidopsis, rice and poplar [22, 72, 73]. We selected 15 putative orthologues of IAMT in different plant species for the phylogenetic analyses (S1 File and S12B Fig). The protein encoded by the *Dca27775* gene was the only member identified in carnation (Fig 8K and Table 3D). The ORF of the *Dca27775* (*DcIAMT*) gene was 1,194 bp long and contained four exons (S11C Fig), which encoded a 397 amino acid protein sharing 73.4% identity and 84.0% similarity with AtIAMT. *DcIAMT* was expressed in the stem cuttings of both the bad-rooting (‘2003 R 8’) and good-rooting (‘2101–02 MFR’) cultivars. On average, the expression levels of *DcIAMT* were higher in ‘2101–02’ *MFR* than in ‘2003 R 8’. Additionally, the expression of *DcIAMT* progressively increased during rooting in the ‘2003 R 8’ cultivar but not in the ‘2101–02 MFR’ (Fig 8K). These results suggest that steady-state levels of non-methylated IAA might be higher in the good-rooting cultivar.

All results considered, it is tempting to speculate that differences in the activities of the auxin conjugating enzymes between the good-rooting and bad-rooting cultivars quantitatively contributes to the observed differences in their rooting behavior due to the direct effect of endogenous active auxin levels in the stem cutting base during rooting.

### Functional validation of auxin homeostasis pathways in carnation stem cuttings

For the functional validation of the auxin homeostasis pathways identified in our phylogenetic analyses, we gathered metabolomic data for several auxin metabolites in mature leaves and in the base of the stem cuttings before rooting (Table 4). High IPyA levels were found only in the mature leaves, and *YUC* gene expression was not found in the stem cutting base during rooting, confirming that the IAA synthesis in carnation stem cuttings was restricted to leaves, as previously suggested [74]. As we did not find the IAM precursor in the leaves or bases of the carnation cuttings, we concluded that the IAM pathway was not relevant for auxin biosynthesis in this species, although it occurs in other species [9, 11]. On the other hand, the highest IAA levels were detected in the basal region of the carnation stem cuttings, suggesting that active basipetal IAA transport from IAA synthesis sites (the leaves) directly contributed to this observation. Additionally, the IAA levels in the stem cutting base were higher in the good-rooting cultivar than in the bad-rooting cultivar, which warrants further investigation. Among the different auxin catabolites measured, only oxIAA and IAA-Asp were detected (Table 4). On the one hand, both were detected only in the stem cutting base and not in the leaves. On the other hand, the IAA-Asp levels were much higher in the bad-rooting cultivar ‘2003 R 8’ than in the good-rooting cultivar ‘2101–02 MFR’. These results indicate a cultivar-dependent regulation of auxin conjugation that requires further investigation.

**Table 4:** Auxin-related metabolites (ng g^-1^ fresh weight) identified in carnation stem cuttings.

	Molecular mass	‘2003 R 8’ mature leaves	‘2003 R 8’ stem cutting base	‘2101–02 MFR’ mature leaves	‘2101–02 MFR’ stem cutting base
**IPyA**	202.050418	1.9±0.6	–	0.1±0.05	–
**IAA**	174.055504	0.8±0.6	6.1±5.5	0.7±0.4	16.2±21.8
**IAA-Asp**	289.082447	–	84.3±73.0	–	25.7±23.6
**IAA-Glc**	336.108327	–	–	–	–
**oxIAA**	190.050418	–	0.4±1.3	–	0.2±0.3
**MeIAA**	188.071154	–	–	–	–

–: not found.

## Conclusions

By combining protein analyses and gene structure studies, we identified putative orthologous genes belonging to the TAA1/TAR, YUC, AMI1, AUX/LAX, PIN, ABCB, GH3, UGT, DAO and IAMT families in carnation. We identified putative gene splicing and allelic variants that had been previously annotated as different genes. We found that the expression profiles of some of these genes involved in auxin transport and catabolism showed contrasting expression patterns in the stem cutting base of two carnation cultivars with different rooting behaviors. In the bad-rooting cultivar ‘2003 R 8’, *DcPIN1a* was highly expressed at 0–6 h and progressively decreased during rooting, while in the good-rooting cultivar, ‘2101–02 MFR’, *DcPIN1a* expression levels were lower at the earlier time points in the bad-rooting cultivar and remained unchanged during rooting. Differential expression profiles between cultivars were found for some genes involved in IAA catabolism, with higher expression levels in the stem cutting base of the bad-rooting cultivar ‘2003 R 8’. Our results suggest that auxin transport and auxin conjugation rates might be higher in the bad-rooting cultivar than in the good-rooting cultivar.

## Supporting information

S1 File Selected genes for phylogenetic analyses. (XLSX)

S1 Fig TAA1/TAR phylogenetic tree. The evolutionary history was inferred by using the Maximum Likelihood method based on the Le Gascuel model [38]. The tree with the highest log likelihood is shown. Initial tree(s) for the heuristic search were obtained automatically by applying Neighbor-Joining and BioNJ algorithms to a matrix of pairwise distances estimated with the most plausible model and then selecting the topology with superior log likelihood value. A discrete Gamma distribution was used to model evolutionary rate differences among sites. The rate variation model allowed for some sites to be evolutionarily invariable. The tree is drawn to scale, with branch lengths measured in the number of substitutions per site. All positions containing gaps and missing data were eliminated. There was a total of 214 positions in the final dataset.
(PDF)

S2 Fig YUC phylogenetic tree. See the legend in S1 Fig for details. There were a total of 104 positions in the final dataset.
(PDF)

S3 Fig AMI1 phylogenetic tree. See the legend in S1 Fig for details. There were a total of 624 positions in the final dataset.
(PDF)

S4 Fig AUX/LAX phylogenetic tree. See the legend in S1 Fig for details. There were a total of 380 positions in the final dataset.
(PDF)

S5 Fig PIN phylogenetic tree. See the legend in S1 Fig for details. There were a total of 224 positions in the final dataset.
(PDF)

S6 Fig ABCB phylogenetic tree. See the legend in S1 Fig for details. There were a total of 250 positions in the final dataset.
(PDF)

S7 Fig Gene structure of carnation ABCB genes. See the legend in S3 Fig for details.
(PDF)

S8 Fig GH3 (group II) phylogenetic tree. See the legend in S1 Fig for details. There were a total of 328 positions in the final dataset.
(PDF)

S9 Fig Gene structure of carnation GH3 (group II) genes. See the legend in S3 Fig for details.
(PDF)

S10 Fig UGT (group L) phylogenetic tree. See the legend in S1 Fig for details. There were a total of 159 positions in the final dataset.
(PDF)

S11 Fig Gene structure of (A) UGT (group L), (B) DAO and (C) IAMT carnation genes. See the legend in S3 Fig for details.
(PDF)

S12 Fig (A) DAO and (B) IAMT phylogenetic trees. See the legend in S1 Fig for details.
(PDF)
