# Supplementary figures and images for: A comprehensive phylogeny of auxin homeostasis genes involved in adventitious root formation in carnation stem cuttings
Source: PLoS One. 2018 Apr 30;13(4):e0196663. doi: 10.1371/journal.pone.0196663 (PMC5927418; doi:10.1371/journal.pone.0196663)

Figure S1

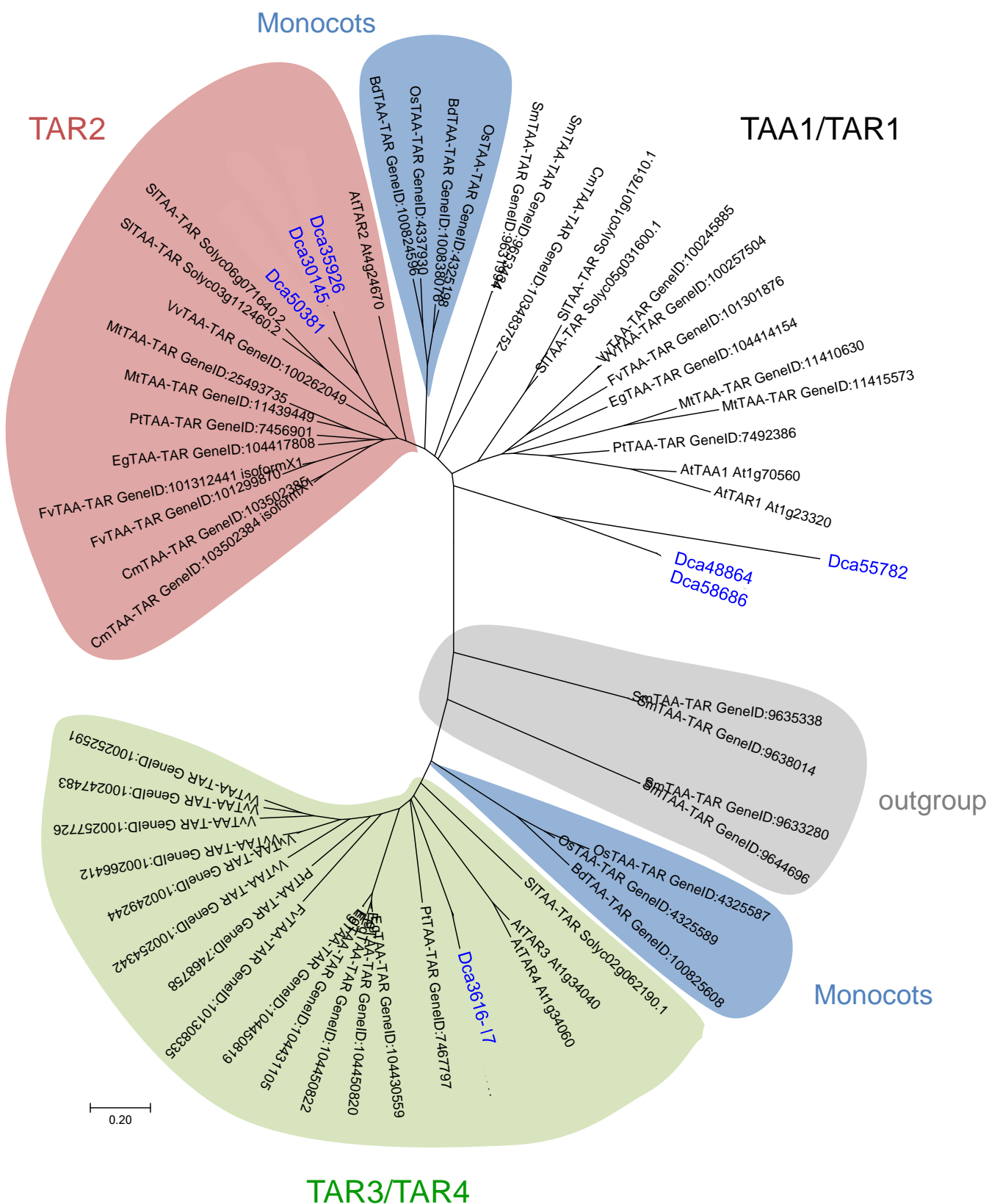

Supplement: S1 Fig — The evolutionary history was inferred by using the Maximum Likelihood method based on the Le Gascuel model [38]. The tree with the highest log likelihood is shown. Initial tree(s) for the heuristic search were obtained automatically by applying Neighbor-Joining and BioNJ algorithms to a matrix of pairwise distances estimated with the most plausible model and then selecting the topology with superior log likelihood value. A discrete Gamma distribution was used to model evolutionary rate differences among sites. The rate variation model allowed for some sites to be evolutionarily invariable. The tree is drawn to scale, with branch lengths measured in the number of substitutions per site. All positions containing gaps and missing data were eliminated. There was a total of 214 positions in the final dataset. (PDF) [file pone.0196663.s002.pdf]

## Figure S2

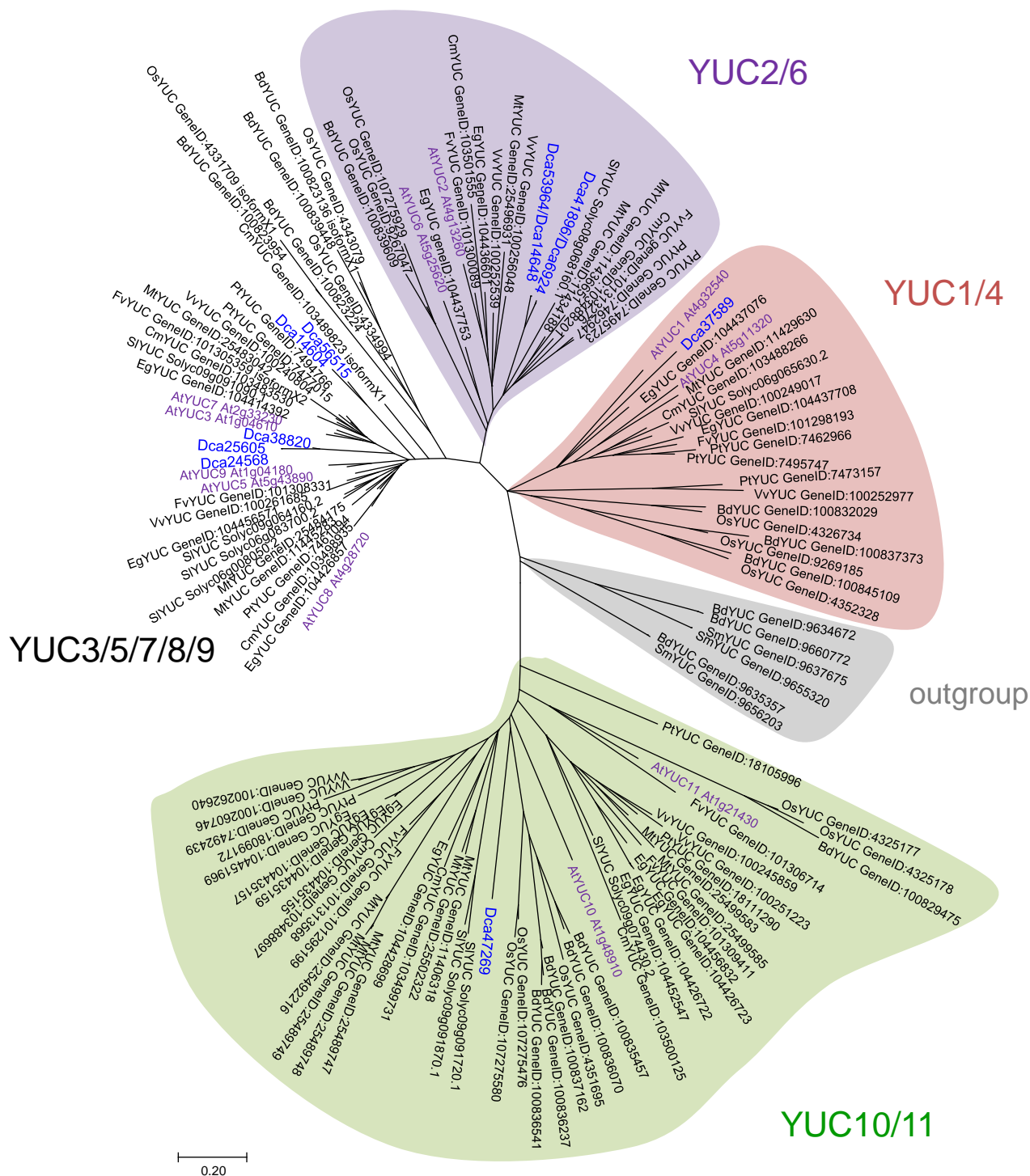

Supplement: S2 Fig — See the legend in S1 Fig for details. There were a total of 104 positions in the final dataset. (PDF) [file pone.0196663.s003.pdf]

Figure S3

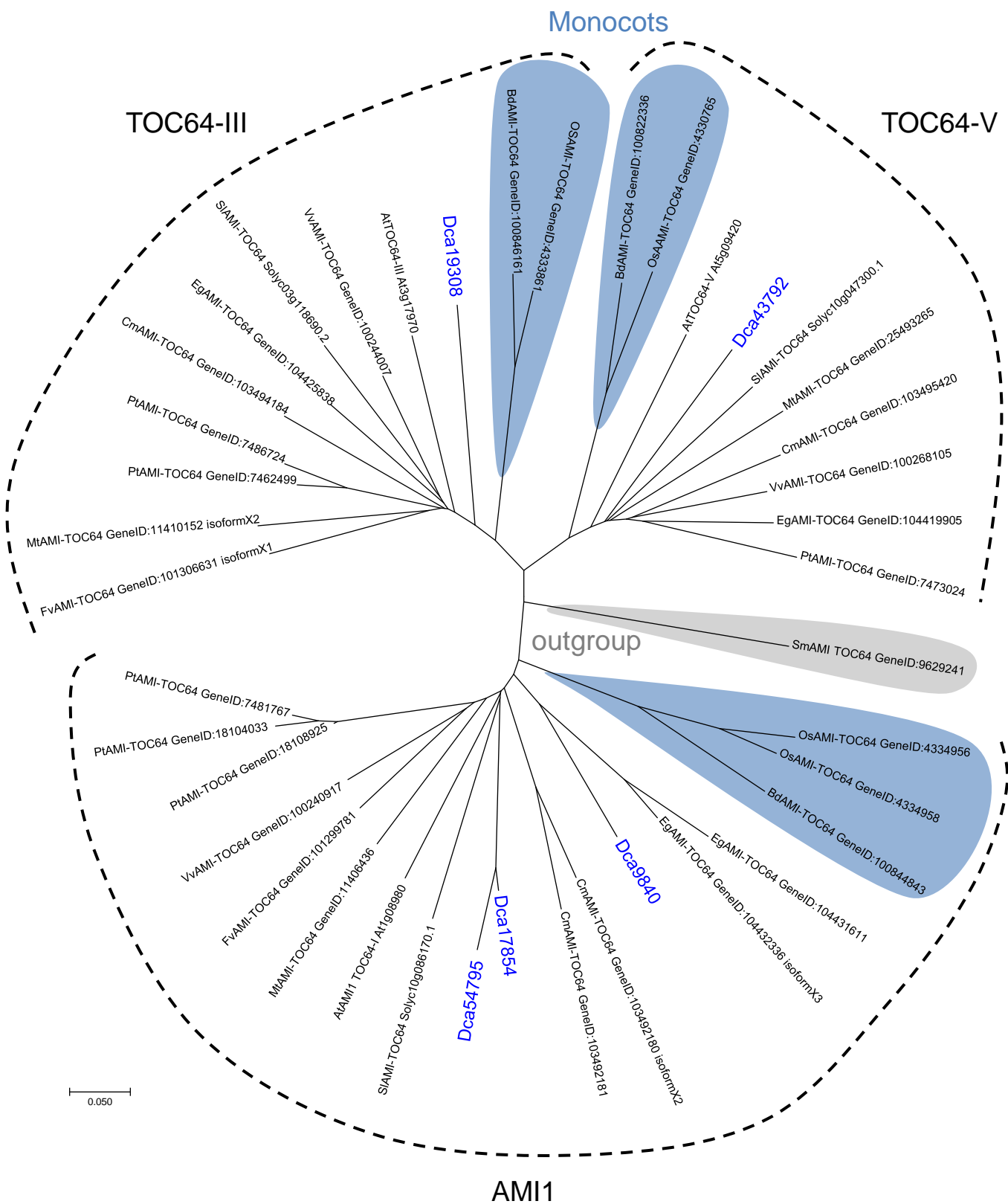

Supplement: S3 Fig — See the legend in S1 Fig for details. There were a total of 624 positions in the final dataset. (PDF) [file pone.0196663.s004.pdf]

Figure S4

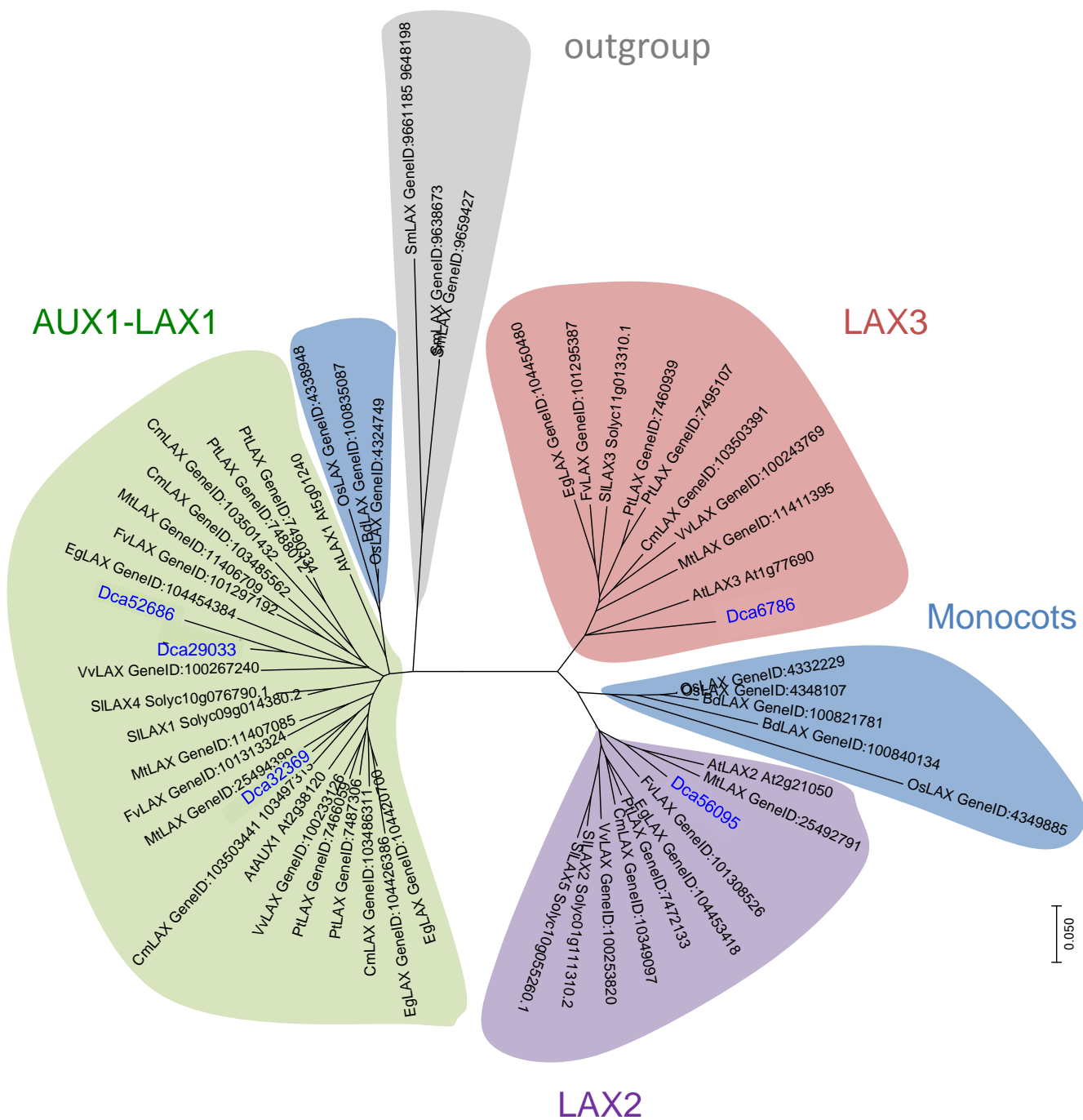

Supplement: S4 Fig — See the legend in S1 Fig for details. There were a total of 380 positions in the final dataset. (PDF) [file pone.0196663.s005.pdf]

Figure S5

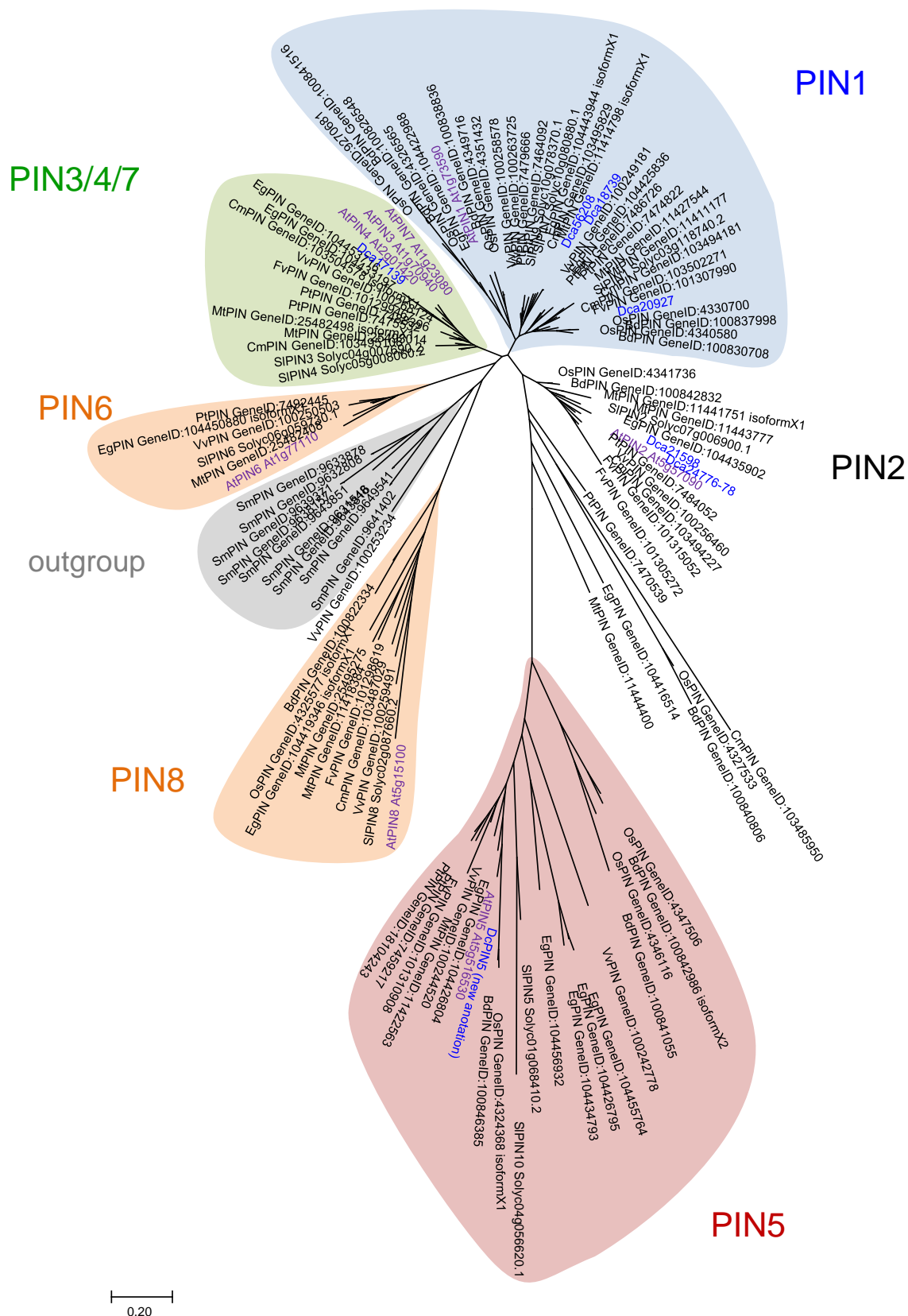

Supplement: S5 Fig — See the legend in S1 Fig for details. There were a total of 224 positions in the final dataset. (PDF) [file pone.0196663.s006.pdf]

Figure S6

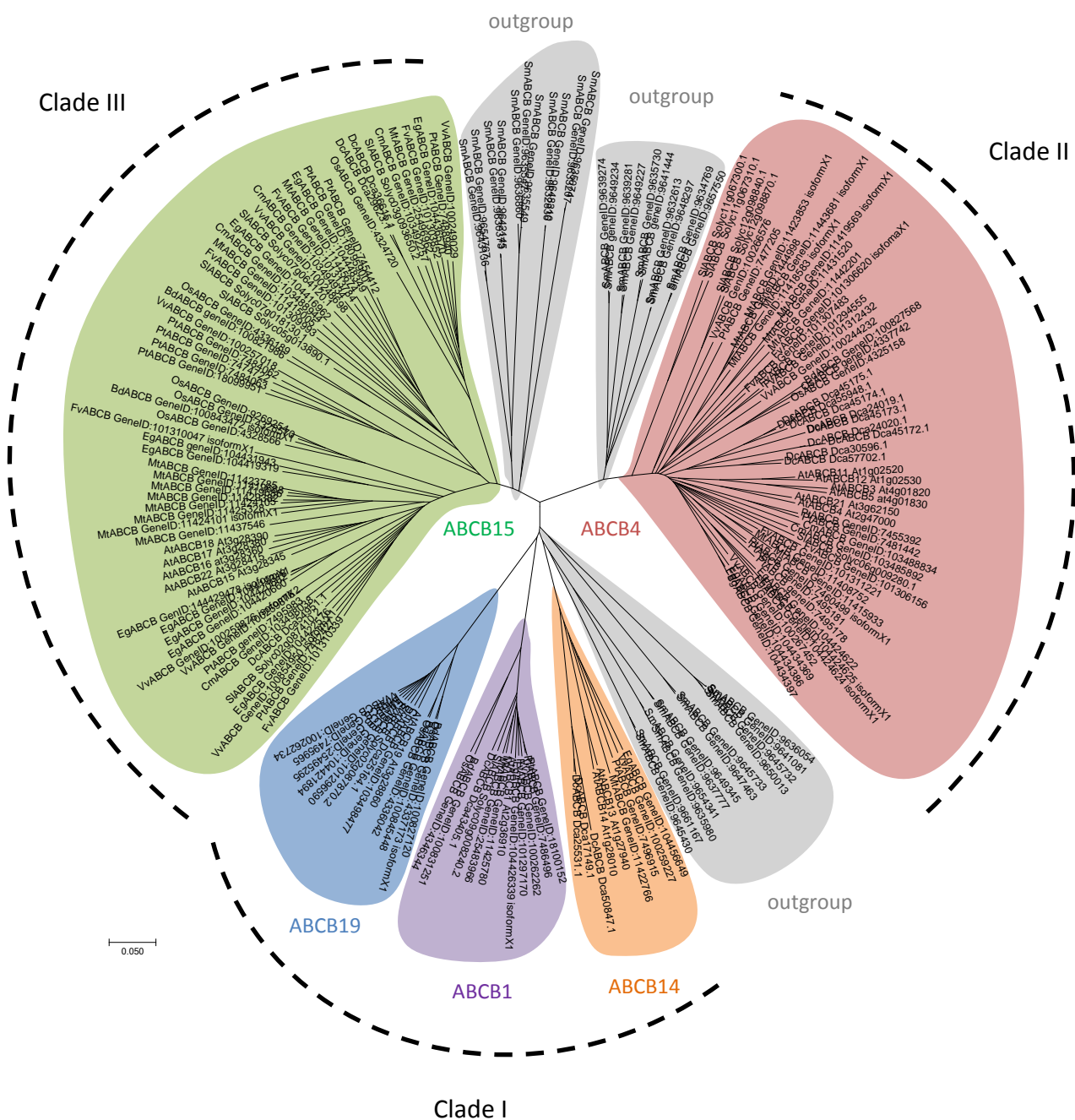

Supplement: S6 Fig — See the legend in S1 Fig for details. There were a total of 250 positions in the final dataset. (PDF) [file pone.0196663.s007.pdf]

Figure S7

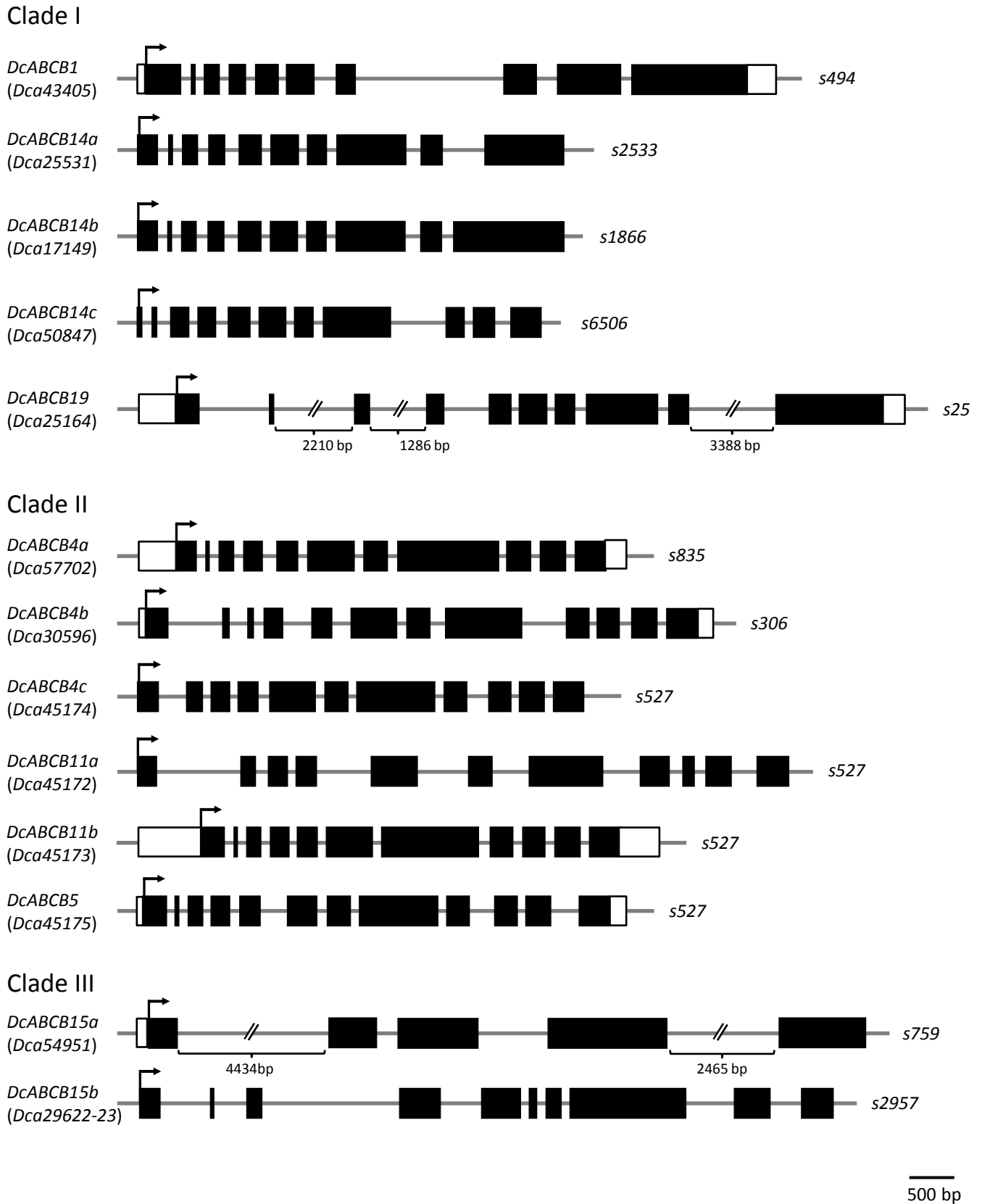

Supplement: S7 Fig — See the legend in S3 Fig for details. (PDF) [file pone.0196663.s008.pdf]

Figure S8

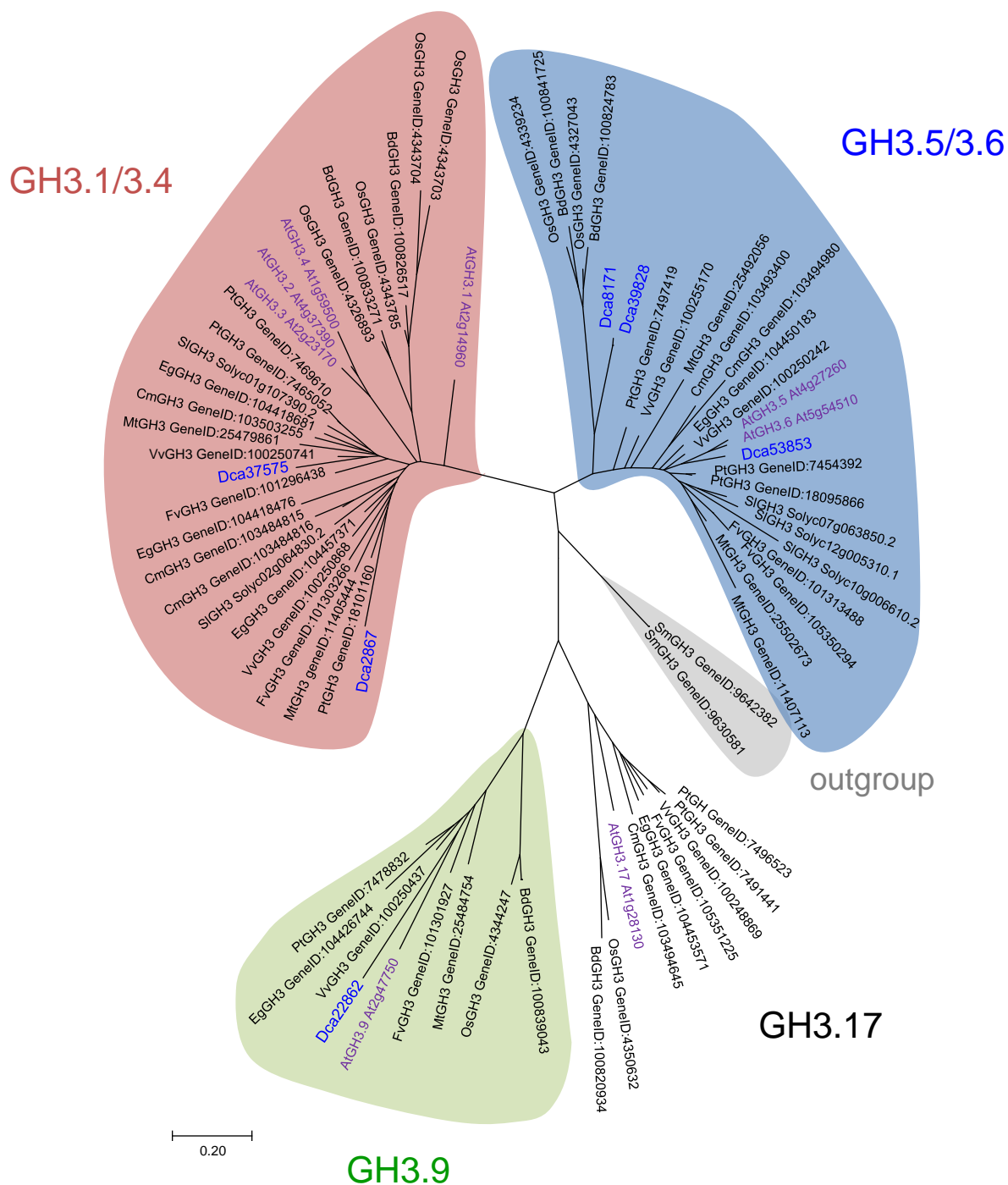

Supplement: S8 Fig — See the legend in S1 Fig for details. There were a total of 328 positions in the final dataset. (PDF) [file pone.0196663.s009.pdf]

Figure S9

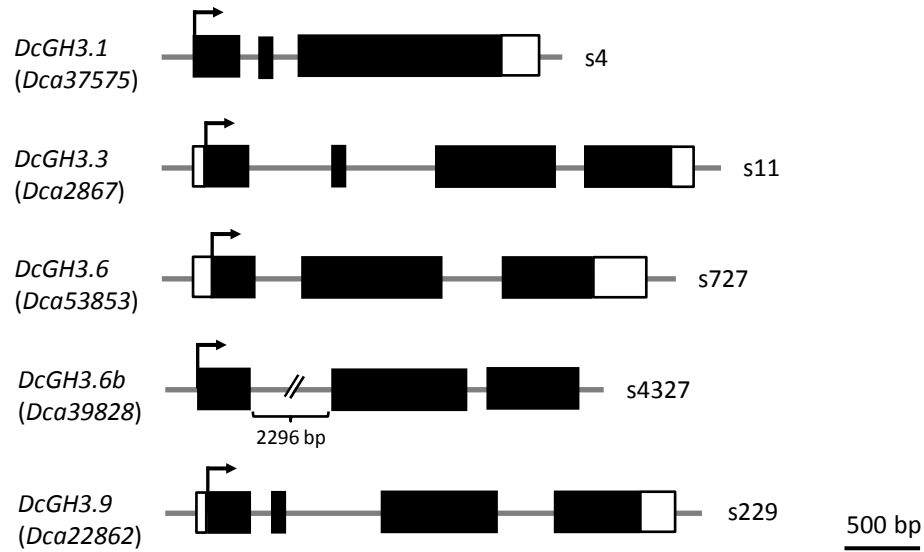

Supplement: S9 Fig — See the legend in S3 Fig for details. (PDF) [file pone.0196663.s010.pdf]

Figure S10

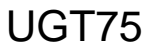

# UGT84

# UGT74

outgroup

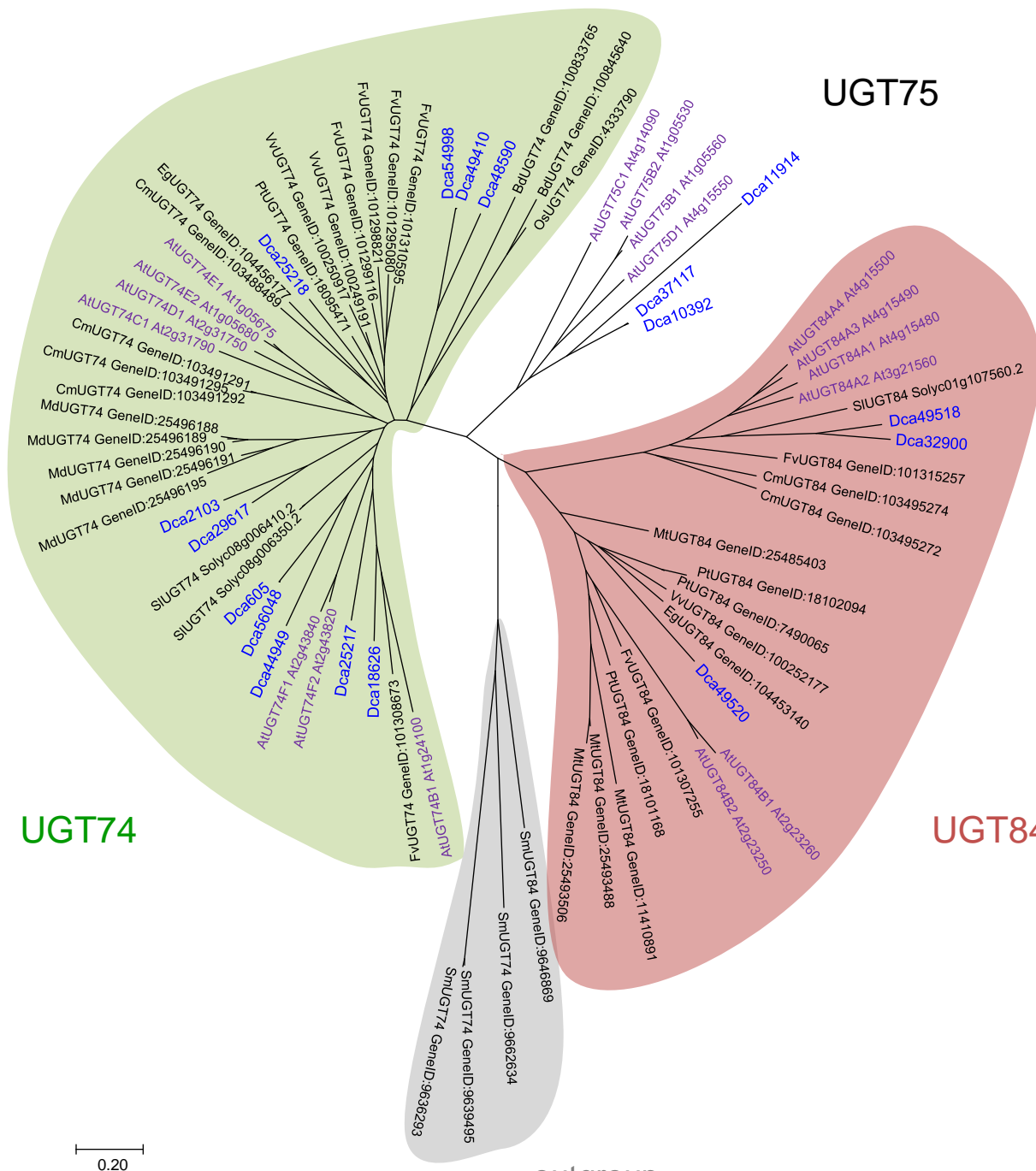

Supplement: S10 Fig — See the legend in S1 Fig for details. There were a total of 159 positions in the final dataset. (PDF) [file pone.0196663.s011.pdf]

Figure S11

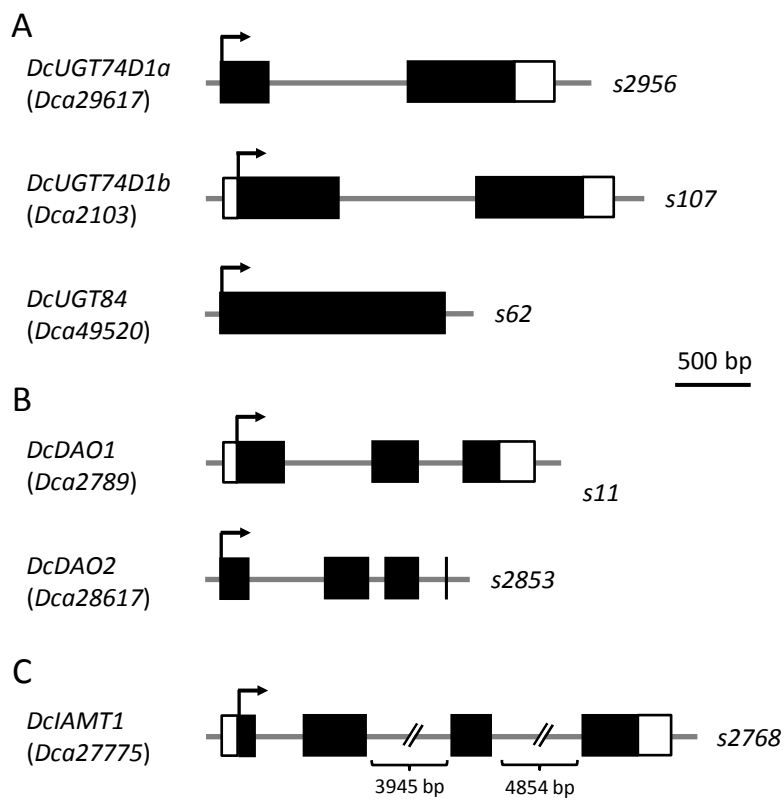

Supplement: S11 Fig — See the legend in S3 Fig for details. (PDF) [file pone.0196663.s012.pdf]

Figure S12

A. DAO

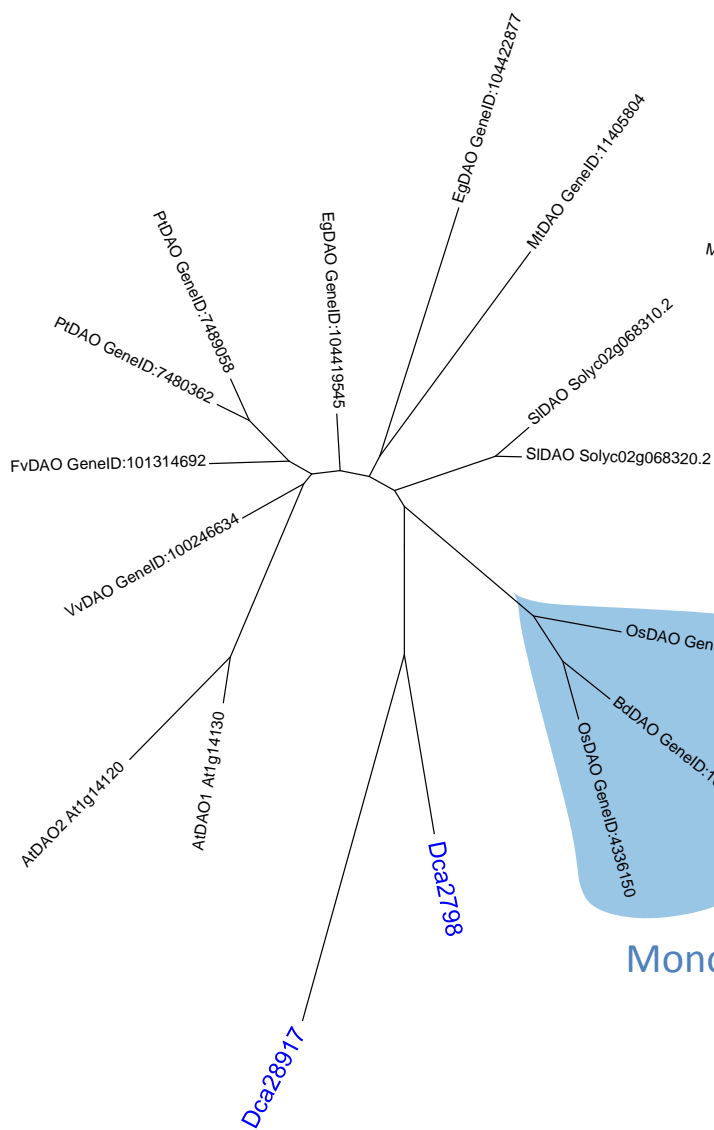

B. IAMT1

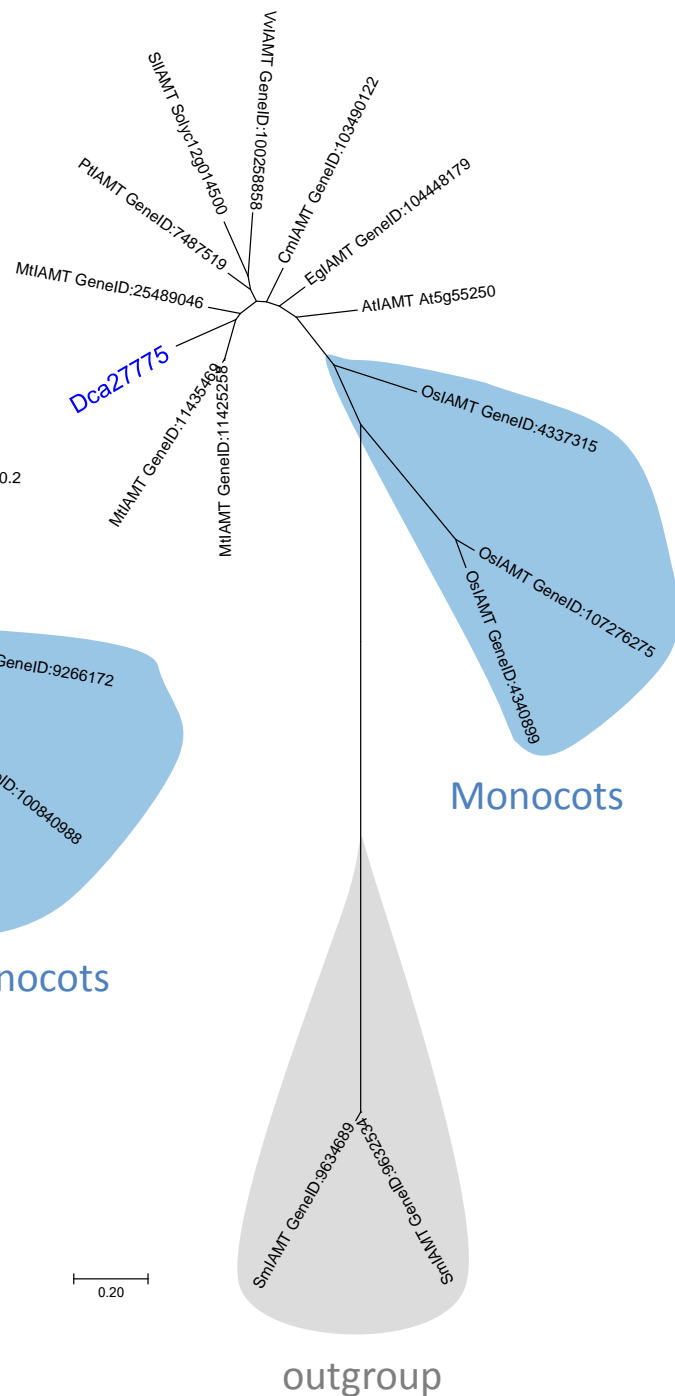

Supplement: S12 Fig — See the legend in S1 Fig for details. (PDF) [file pone.0196663.s013.pdf]
